# PDP-1 Links the TGF-β and IIS Pathways to Regulate Longevity, Development, and Metabolism

**DOI:** 10.1371/journal.pgen.1001377

**Published:** 2011-04-21

**Authors:** Sri Devi Narasimhan, Kelvin Yen, Ankita Bansal, Eun-Soo Kwon, Srivatsan Padmanabhan, Heidi A. Tissenbaum

**Affiliations:** 1 Program in Gene Function and Expression, University of Massachusetts Medical School, Worcester, Massachusetts, United States of America; 2 Program in Molecular Medicine, University of Massachusetts Medical School, Worcester, Massachusetts, United States of America; Stanford University Medical Center, United States of America

## Abstract

The insulin/IGF-1 signaling (IIS) pathway is a conserved regulator of longevity, development, and metabolism. In *Caenorhabditis elegans* IIS involves activation of DAF-2 (insulin/IGF-1 receptor tyrosine kinase), AGE-1 (PI 3-kinase), and additional downstream serine/threonine kinases that ultimately phosphorylate and negatively regulate the single FOXO transcription factor homolog DAF-16. Phosphatases help to maintain cellular signaling homeostasis by counterbalancing kinase activity. However, few phosphatases have been identified that negatively regulate the IIS pathway. Here we identify and characterize *pdp-1* as a novel negative modulator of the IIS pathway. We show that PDP-1 regulates multiple outputs of IIS such as longevity, fat storage, and dauer diapause. In addition, PDP-1 promotes DAF-16 nuclear localization and transcriptional activity. Interestingly, genetic epistasis analyses place PDP-1 in the DAF-7/TGF-β signaling pathway, at the level of the R-SMAD proteins DAF-14 and DAF-8. Further investigation into how a component of TGF-β signaling affects multiple outputs of IIS/DAF-16, revealed extensive crosstalk between these two well-conserved signaling pathways. We find that PDP-1 modulates the expression of several insulin genes that are likely to feed into the IIS pathway to regulate DAF-16 activity. Importantly, dysregulation of IIS and TGF-β signaling has been implicated in diseases such as Type 2 Diabetes, obesity, and cancer. Our results may provide a new perspective in understanding of the regulation of these pathways under normal conditions and in the context of disease.

## Author Summary

Cells in the body respond to a variety of on/off signals that are relayed in a defined spatial and temporal manner. These signals influence several processes such as growth, fat storage, and the repair of damaged molecules. As humans age, the onset of diseases such as Type 2 Diabetes, obesity, and cancer often results from an imbalance in the levels of on/off signals in the cell. The insulin/IGF-1 signaling pathway is an important regulator of longevity, development, and metabolism across phylogeny. While the protein kinases that activate this pathway have been well studied, less is known about the protein phosphatases that tune down the signals. The roundworm *C. elegans* has been an excellent model system to study the role of insulin/IGF-1 signaling in the aging process. Here, we identify a new phosphatase that negatively regulates the insulin/IGF-1 pathway to enhance longevity and stress-resistance. Interestingly, the phosphatase achieves this function by tuning down the activity of a conserved TGF-β pathway, a pathway important for development. By reducing TGF-β pathway activity, this phosphatase decreases expression of insulin molecules that may stimulate the insulin/IGF-1 pathway. Our studies not only unravel a new regulator of these pathways, but also point to how they are more linked than previously thought. Both insulin/IGF-1 and TGF-β signaling have been implicated in age-associated diseases, and understanding their connection will provide us with potential therapeutic avenues.

## Introduction

Insulin/IGF-1 signaling (IIS) is a conserved neuroendocrine pathway that regulates longevity, development and energy metabolism across phylogeny [1], [2]. In the roundworm *Caenorhabditis elegans (C. elegans)*, activation of the DAF-2 insulin/IGF-1 receptor tyrosine kinase intiates an AAP-1/AGE-1 PI 3-kinase signaling cascade involving the downstream serine/threonine kinases PDK-1, AKT-1, and AKT-2 [3]–[7]. Activated AKT-1 and AKT-2 phosphorylate DAF-16, the single Forkhead Box O (FOXO) family transcription factor homolog in *C. elegans* [8]. Phosphorylation of DAF-16 results in its inactivation and sequestration in the cytosol [9], [10]. Under low signaling conditions, DAF-16 translocates into the nucleus, where it can transactivate/repress hundreds of target genes [9]–[13].

The dauer is an alternative survival stage that worms can enter upon poor environmental conditions such as crowding [14]. Mutations in the kinases upstream of DAF-16 such as *daf-2*, *age-1*, *pdk-1*, *akt-1* and *akt-2* result in an increase in lifespan, dauer formation, fat storage and/or stress resistance, and loss-of-function mutations in *daf-16* completely suppress these phenotypes [15]–[18]. In addition to the IIS pathway, dauer formation in *C. elegans* is also regulated by the DAF-7/TGF-β-like signaling pathway [19]–[21]. Activation of TGF-β signaling is achieved through binding of the DAF-7 BMP-like ligand to the DAF-1/DAF-4, the Type I/II receptors, which phosphorylate and activate the downstream receptor-associated SMAD (R-SMAD) proteins DAF-8 and DAF-14, presumably through a conserved SSXS phosphorylation motif that has been shown to be important for R-SMAD activation in mammals [22]–[24]. Upon activation, R-SMADs can associate with a Co-SMAD to regulate the transcription of hundreds of genes [23], [25]. In *C. elegans*, DAF-8 and DAF-14 act to antagonize the transcriptional activity of the DAF-3 Co-SMAD and the DAF-5 SNO-SKI repressor [22], [24], [26]–[29]. Reduction of function mutations in *daf-7*, *daf-1*, *daf-4*, *daf-8* and *daf-14* show temperature-sensitive constitutive dauer formation and mutations in *daf-3* and/or *daf-5* completely suppress this phenotype [19], [21], [30]. Genetic epistasis studies have suggested that the TGF-β pathway acts in a parallel manner with IIS to modulate dauer formation [31]–[33].

The PTEN lipid phosphatase homolog DAF-18, which antagonizes signaling at the level of AGE-1/PI 3-kinase, is a major negative regulator of IIS. In contrast to the kinases in this pathway, loss-of-function mutations in *daf-18* reduces lifespan, fat storage, dauer formation and stress resistance [32], [34]–[39]. Besides DAF-18, few negative modulators of the pathway have been identified. In particular, less is known about serine/threonine phosphatases that counterbalance kinase activity in the IIS pathway. We recently performed a directed RNA interference (RNAi) screen for serine/threonine phosphatases that regulate *C. elegans* IIS using dauer formation as an output [39]. We identified the PP2A regulatory subunit PPTR-1 as an important regulator of AKT-1 dephosphorylation as well as DAF-16-dependent phenotypes [39]. Here we characterize another candidate from this screen, *pdp-1*, as a positive regulator of dauer formation. PDP-1 is homologous to pyruvate dehydrogenase phosphatase (PDP) in higher organisms, an enzyme that positively regulates the pyruvate dehydrogenase enzyme complex (PDHc). RNAi of the other components of PDHc do not result in changes in dauer formation. Interestingly, we report that although PDP-1 is a robust modulator of multiple IIS-regulated processes as well as DAF-16 activity, genetic epistasis studies place *pdp-1* in the DAF-7/TGF-β pathway. Through this study, we find that IIS and TGF-β signaling are more tightly connected than previously suggested, with distinct roles for the Co-SMAD DAF-3 in modulating the IIS pathway. Our data suggests that PDP-1 modulates the gene expression of several insulins, and that insulins may be a potential mediator of the crosstalk between these two pathways.

## Results

### *C. elegans* PDP-1 regulates *daf-2* dauer formation independent of PDH

**Figure 1:**
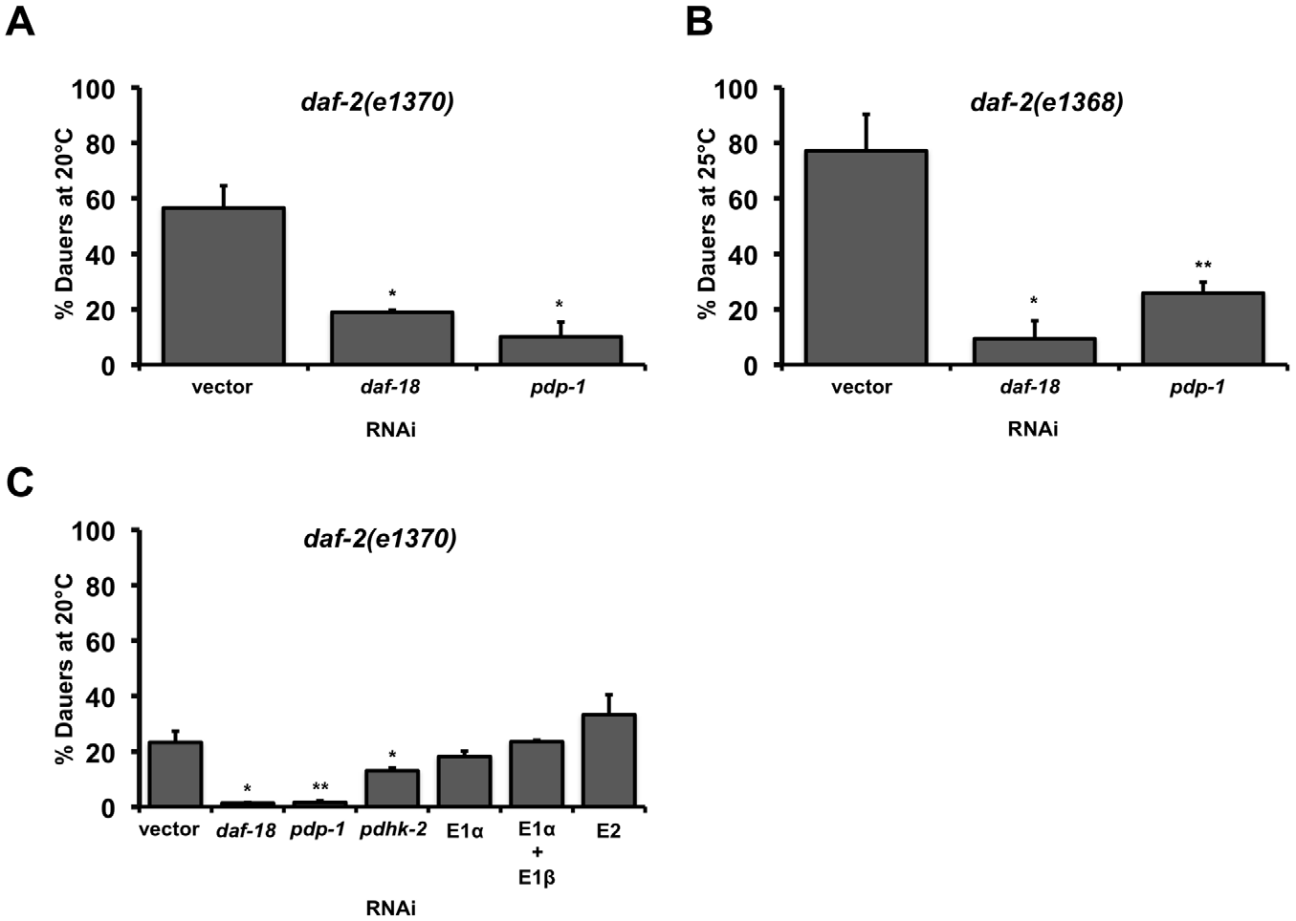
PDP-1 regulates *daf-2* dauer formation independent of the PDHc. Error bars indicate the standard deviation among the different RNAi plates within one experiment. Data shown are from one representative experiment. (A) *pdp-1* RNAi suppresses *daf-2(e1370)* dauer formation similar to *daf-18* RNAi. Dauer formation of *daf-2(e1370)* is 56.5±8.0% (n = 278) on vector RNAi, 18.9±0.8% (n = 79) on *daf-18* RNAi (p<0.05) and 10.5±5.3% (n = 293) on *pdp-1* RNAi (p<0.05). (B) *pdp-1* RNAi suppresses dauer formation of *daf-2(e1368)* worms similar to *daf-18* RNAi. Dauer formation of *daf-2(e1368)* is 77.1±13.2% dauers (n = 297) on vector RNAi, *daf-2(e1368)* worms form only 9.4±6.4% (n = 258) dauers on *daf-18* RNAi (p<0.06) and 25.9±3.9% (n = 636) dauers on *pdp-1* RNAi (p<0.05). (C) RNAi of other components of the PDHc including the E1α subunit does not affect *daf-2(e1370)* dauer formation. Dauer formation of *daf-2(e1370)* on PDHc RNAi is 23.3±4.1% (n = 282) on vector RNAi, 1.3±0.2% (n = 219) on *daf-18* RNAi (p<00.04), 1.6±0.6% (n = 185) on *pdp-1* RNAi (p<0.03), 13.1±1.0% (n = 233) on *pdhk-2* on RNAi (p<0.05), 18.2±2.0% (n = 193) on E1α RNAi, 23.5±0.5% (172) on a combination of E1α and E1β RNAi and 33.3±7.1% (n = 25) on E2 RNAi.

Our RNAi screen was designed to identify serine/threonine phosphatases that modulated dauer formation of *daf-2(e1370)*, a non-null, temperature-sensitive mutant of the *C. elegans* insulin/IGF-1 receptor gene, *daf-2* [39]. We were particularly interested in phosphatases that would negatively regulate IIS similar to DAF-18/PTEN, and for all RNAi based assays described below, *daf-18* RNAi was used as a positive control [39]. From this screen, we identified *pdp-1* as a modulator of *daf-2(e1370)* dauer formation (Figure 1A and Figure S2). BLAST analyses using amino acid sequence revealed that PDP-1 is homologous to fly and mammalian PDP (∼52% positive and ∼38% identical). *pdp-1* RNAi significantly reduces dauer formation of *daf-2(e1370)* worms, similar to *daf-18* RNAi (Figure 1A and Figure S2). This phenotype is not allele-specific, as *pdp-1* RNAi results in suppression of dauer formation in a second allele of *daf-2, daf-2(e1368)* (Figure 1B and Figure S2). Similar to the results with the RNAi, a mutation in *pdp-1* also affects dauer formation - *pdp-1(tm3734)*; *daf-2(e1370)* double mutants form significantly fewer dauers when compared to the *daf-2(e1370)* parental strain (Figure S2).

Given its homology to PDP in higher organisms, we wondered whether the effect of *pdp-1* knockdown on *daf-2* dauer formation was a consequence of modulating the activity of the PDHc. The PDHc is a multi-subunit enzyme complex consisting of three major enzymes: E1 pyruvate dehydrogenase, E2 dihydrolipoyl acetyltransferase and E3 dihydrolipoyl dehydrogenase that regulate energy metabolism [40]. PDHc converts pyruvate to acetyl-coA, which can either enter the TCA cycle or be used for fatty acid synthesis. In mammals, regulation of PDHc activity is primarily achieved through reversible phosphorylation/dephosphorylation of the E1α subunit by pyruvate dehydrogenase kinase (PDHK) and PDP, with phosphorylation inactivating the enzyme complex [40]. All of the components of the PDH complex have conserved *C. elegans* homologs, encoded by the genes T05H10.6 (E1α), C04C3.3 (E1β), F23B12.5 (E2), LLC1.3 (E3), *pdhk-2* (PDHK) and *pdp-1* (PDP).

To test whether modulation of PDHc activity affects *daf-2* dauer formation, we grew *daf-2(e1370)* worms on PDHc RNAi. Quantification the RNAi efficiency of the PDHc components revealed that we achieved 60–90% knockdown (Figure S1). To our surprise, RNAi of the E1α subunit had no effect on *daf-2* dauer formation, while *pdp-1* RNAi resulted in dauer suppression (Figure 1C and Figure S2). In addition, RNAi of either the other E1 subunit E1β, or the E2 subunit, did not affect *daf-2* dauer formation (Figure 1C and Figure S2). Knockdown of the E3 subunit resulted in lethality (data not shown). Interestingly, *pdhk-2* RNAi resulted in slight suppression *daf-2(e1370)* dauer formation but had no effect on dauer formation of *daf-2(e1368)* mutants (Figure 1C and Figure S2). Therefore *pdhk-2* modulates the IIS pathway in an allele-specific manner and we did not perform further characterization of this gene.

To further evaluate the components of the PDH complex, we examined their expression patterns. The expression pattern of PDP-1 does not completely overlap with that of the E1 and E2 subunits of PDHc (Figure S3). PDP-1 expression was enriched in the head and tail neurons, head muscle and the intestine. We did not observe any expression in the pharynx. In contrast, the expression of the E1 and E2 subunits, was observed throughout the body of the worm and was significantly enriched in the pharynx. Taken together, PDP-1 modulates *daf-2* dauer formation and this function is likely to be independent of its role in regulating the PDHc.

### PDP-1 regulates multiple outputs of the IIS pathway

**Figure 2:**
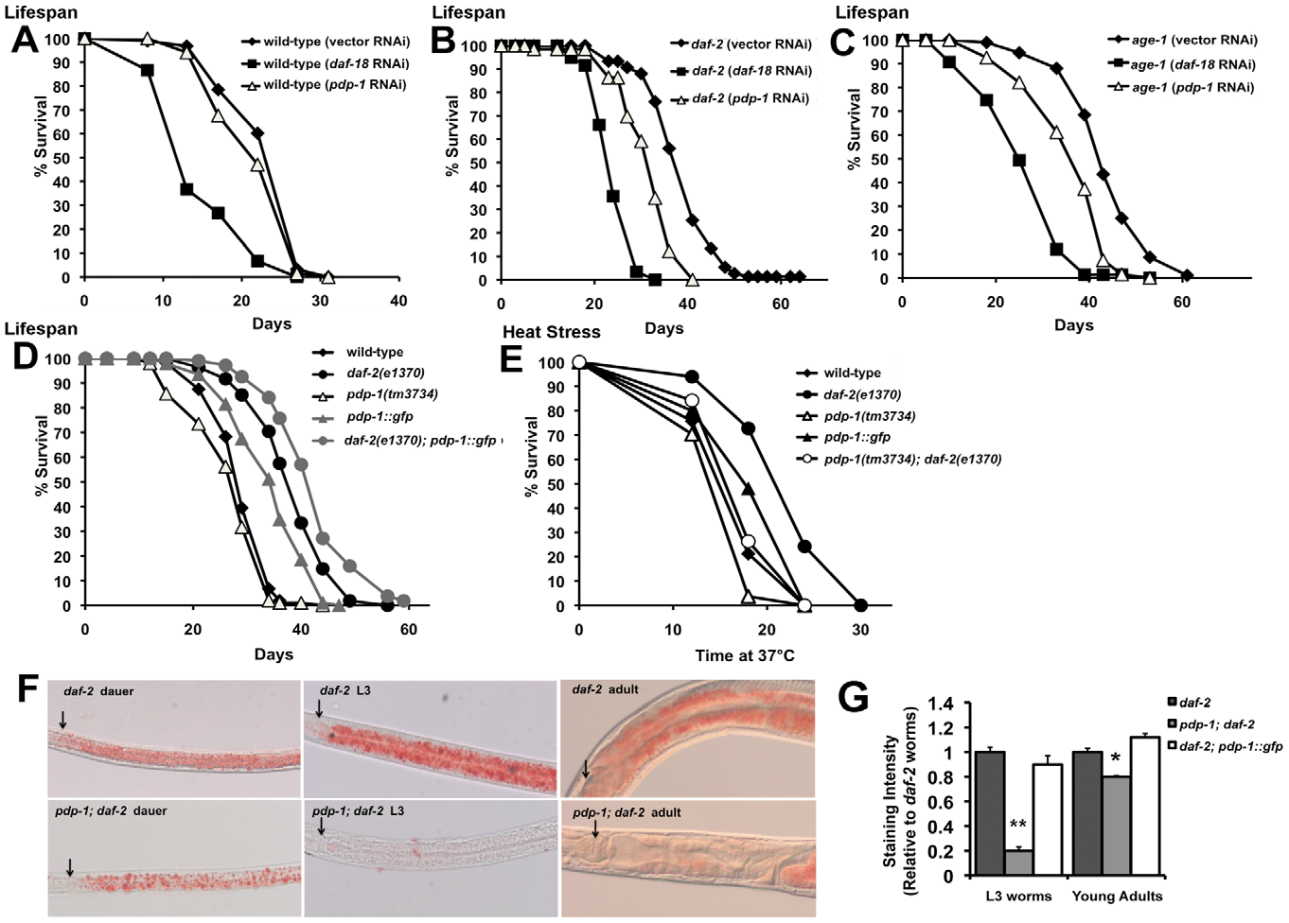
PDP-1 regulates multiple outputs of the IIS pathway. Data shown are from one representative experiment. (A) *pdp-1* RNAi does not significantly reduce the lifespan of wild-type worms. Mean lifespan of wild-type worms is 23.8±0.5 days (n = 93) on vector RNAi, 14.5±0.9 days (n = 34) on *daf-18* RNAi (p<0.0001) and 22.6±0.6 days (n = 68) on *pdp-1* RNAi (p<0.08). (B) The increased lifespan of *daf-2(e1370)* worms is reduced by *pdp-1* RNAi. Mean lifespan of *daf-2(e1370)* worms is 38.9±0.9 days (n = 75) on vector RNAi, 24.5±0.5 days (n = 59) on *daf-18* RNAi (p<0.0001) and 31.7±0.8 days (n = 66) on *pdp-1* RNAi (p<0.0001). (C) *pdp-1* RNAi reduces the increased lifespan of *age-1(hx546)* mutants. Mean lifespan of *daf-2(e1370)* worms is 42.8±0.8 days (n = 84) on vector RNAi, 28.0±0.9 days (n = 81) on *daf-18* RNAi (p<0.0001) and 36.5±1.0 days (n = 67) on *pdp-1* RNAi (p<0.0001). (D) *pdp-1* overexpression increases the lifespan of wild-type and *daf-2(e1370)* worms while *pdp-1* mutants live slightly shorter than wild-type animals. Mean lifespan of wild-type worms is 29.4±0.5 days (n = 104), *pdp-1(tm3734)* mutants was 27.1±0.7 days (n = 98), p<0.05, *pdp-1::gfp* mutants is 34.5±0.8 days (n = 92) p<0.0001, *daf-2(e1370)* is 38.7±0.7 days (n = 108) and *daf-2(e1370)*; *pdp-1::gfp* is 42.8±0.7 days (n = 105) days p<0.0001. (E) PDP-1 regulates thermotolerance. Mean survival of wild-type worms is 18.3±0.7 hours (n = 37), *pdp-1(tm3734)* mutants is 17.1±0.8 hours (n = 27) p<0.2, *pdp-1::gfp* worms is 19.7±0.9 days (n = 25) p<0.09, *daf-2(e1370)* worms is 21.6±0.6 hours (n = 30) and *pdp-1(tm3734)*; *daf-2(e1370)* worms is 18.6±0.9 hours (n = 19), p<0.0007). (F) Oil Red O staining reveals that *pdp-1(tm3734)*; *daf-2(e1370)* worms store less fat than *daf-2* worms across different stages in the worm life cycle: dauers (left), L3 worms (middle) and adults (right). Arrows indicate the lower bulb of the pharynx. (G) Quantification of Oil Red O staining in L3 and young adults of *daf-2(e1370)*, *pdp-1(tm3734)*; *daf-2(e1370)* and *daf-2(e1370)*; *pdp-1::gfp* worms. A mutation in *pdp-1* significantly reduces *daf-2(e1370)* fat storage in both, L3s (p<0.0001) and young adults (p<0.01). In adult worms, *daf-2(e1370)*; *pdp-1::gfp* worms store slightly more fat than *daf-2(e1370)* not in younger L3 animals (p<0.02).

In addition to dauer formation, the IIS pathway also regulates longevity, stress resistance and fat storage [17], [18]. Mutations in *daf-2* and *age-1* result in a significant extension in lifespan, enhanced resistance to various stresses and increased fat storage [7], [35], [41]–[44]. These phenotypes are suppressed by loss-of-function mutations in *daf-18* and *daf-16* [32], [34], [35], [39]. We therefore investigated whether dosage modulation of *pdp-1* would affect additional outputs of the pathway. We first tested the role of PDP-1 in regulating lifespan (Figure 2 and Figure S4). The lifespan of wild-type worms was not affected by *pdp-1* RNAi and slightly reduced by a mutation in *pdp-1* (Figure 2A and 2D). In contrast, the mean and maximal lifespan of long-lived *daf-2(e1370)* and *age-1(hx546)* mutants was significantly reduced by *pdp-1* RNAi (Figure 2B and 2C). Similarly, *pdp-1(tm3734)*; *daf-2(e1370)* double mutants lived significantly shorter than the parental *daf-2(e1370)* strain (Figure S4).

To examine the effect of increased dosage of *pdp-1*, we generated transgenic worms bearing a translational fusion containing *pdp-1* fused to *gfp* and driven by its own promoter (*pdp-1::gfp*). In addition, we also crossed the *pdp-1::gfp* worms to *daf-2(e1370)* mutants to generate the *daf-2(e1370)*; *pdp-1::gfp* strain. Overexpression of *pdp-1* results in a significant extension in lifespan compared to wild-type worms (Figure 2D and Figure S4). Interestingly, *pdp-1* overexpression further extends the lifespan of *daf-2(e1370)* mutants (Figure 2B and Figure S4). In both of these cases, the increased lifespan was suppressed by *daf-16* RNAi (Figure S5). Therefore, dosage modulation of *pdp-1* regulates lifespan in a DAF-16 dependent manner.

Next, we asked if PDP-1 modulated additional outputs of the IIS signaling pathway. We first tested whether PDP-1 regulates stress resistance by assaying the survival of *pdp-1* mutants and transgenic animals when exposed to heat stress at 37°C (Figure 2E and Figure S7). Dosage modulation of *pdp-1* affects the response to heat stress, with a *pdp-1* mutation decreasing and *pdp-1* overexpression slightly enhancing thermotolerance (Figure 2E). Importantly a *pdp-1* mutation drastically reduced the thermotolerance of *daf-2* mutants (Figure 2E).

To examine the role of *pdp-1* in regulating fat storage, we used both Oil Red O [45] and Sudan Black [7] staining (Figure 2F and 2G and Figure S7). *pdp-1* mutants had similar levels of fat compared to wild-type worms, while overexpression of *pdp-1* slightly enhanced fat storage (Figure S7). In contrast, a *pdp-1* mutation drastically reduced the increased fat of *daf-2(e1370)* mutants (Figure 2F and 2G and Figure S7). This was observed in dauers, larval stage 3 (L3) animals and adults, suggesting that PDP-1 is an important regulator of fat storage in *daf-2* mutants. We did observe any further enhancement of the increased fat storage in the *daf-2(e1370)*; *pdp-1::gfp* worms (Figure S7). Importantly, the increased fat storage of *pdp-1::gfp* and *daf-2(e1370)*; *pdp-1::gfp* worms was suppressed by *daf-16* RNAi, similar to *daf-2* mutants (Figure S7). Thus, PDP-1 modulates all four well-characterized outputs of the IIS pathway.

In addition to these phenotypes, *pdp-1(tm3734)* mutants exhibit a slow movement phenotype, which we quantified using locomotion assays (Figure S6). This slow movement was rescued by the *pdp-1::gfp* transgene. In addition, we performed brood size analysis of wild-type, *pdp-1(tm3734)* mutants, *daf-2(e1370)* mutants, and *pdp-1(tm3734)*; *daf-2(e1370)* double mutants (Figure S6). *pdp-1(tm3734)* worms showed a slight decrease in the number of progeny compared to wild-type worms. However, when compared to *daf-2* mutants, only 5% of the *pdp-1(tm3734)*; *daf-2(e1370)* eggs yielded progeny (Figure S6). *daf-2* mutants have a slightly reduced brood size [46], [47], and a mutation in *pdp-1* severely enhances this phenotype . Taken together, PDP-1 regulates multiple outputs of IIS and acts as a negative regulator the pathway, similar to DAF-18/PTEN.

### PDP-1 positively regulates DAF-16

**Figure 3:**
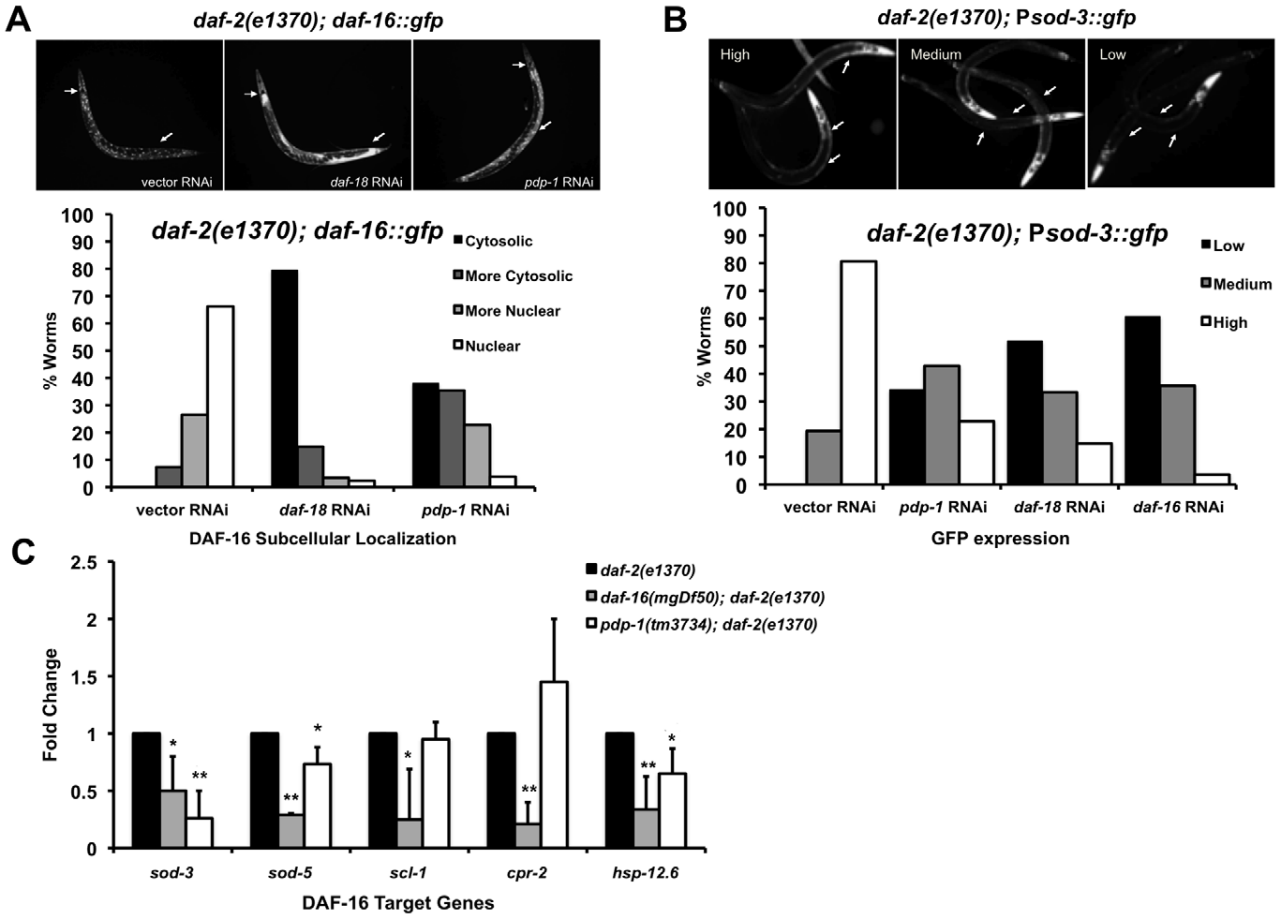
PDP-1 regulates DAF-16 nuclear localization and transcriptional activity. (A) DAF-16::GFP localization visualized in *daf-2(e1370)*; *daf-16::gfp* worms on vector, *daf-18* and *pdp-1* RNAi (top panel, 100× magnification) and quantification of DAF-16::GFP nuclear-cytosolic localization (lower panel). Data shown are from one representative experiment. (n = 68 on vector RNAi, n = 88 on *daf-18* RNAi and n = 79 on *pdp-1* RNAi). (B) Representative images of high, medium and low GFP expression in *daf-2(e1370)*; *Psod-3::gfp* worms (top panel, 100× magnification). Quantification of GFP expression in *daf-2(e1370)*;*Psod-3::gfp* worms on vector, *daf-18*, *pdp-1* and *daf-16* RNAi (Lower panel). Data shown are from one representative experiment (n = 31 on vector RNAi, n = 35 on *pdp-1* RNAi, n = 27 on *daf-18* RNAi and n = 28 on *daf-16* RNAi). (C) Levels of known DAF-16 targets are reduced in *pdp-1(tm3734)*; *daf-2(e1370)* worms when compared to *daf-2(e1370)* worms. Data shown is an average of three independent repeats. * p<0.05, **p<0.01.

The FOXO transcription factor DAF-16 is the major target of the *C. elegans* IIS pathway [2], [48]. Under conditions of reduced IIS, DAF-16 is able to translocate into the nucleus, where it regulates the expression of hundreds target genes [12], [13], [49], [50]. We therefore asked whether PDP-1 modulates DAF-16 subcellular localization as well as activity (Figure 3A and Figure S8). *daf-2(e1370)*; *daf-16::gfp* worms were grown on vector, *daf-18* and *pdp-1* RNAi, and DAF-16 nuclear/cytosolic localization was visualized using fluorescence microscopy and quantified. Throughout the body of the worm, while DAF-16::GFP was mostly nuclear on vector RNAi, its localization was enriched in the cytosol on *pdp-1* RNAi, similar to *daf-18* RNAi (Figure 3A and Figure S8).

The gene superoxide dismutase 3 *(sod-3)* is a direct DAF-16 target [11]. To test whether PDP-1 modulates transcriptional activity of DAF-16, we used a *Psod-3::gfp* reporter strain in a *daf-2(e1370)* background [51]. *daf-2(e1370)*; *Psod-3::gfp* worms were grown on vector, *pdp-1*, *daf-18* and *daf-16* RNAi and GFP expression was visualized using fluorescence microscopy and scored as low, medium or high (Figure 3B and Figure S8). GFP expression was markedly lower on *pdp-1* RNAi compared to vector RNAi, similar to *daf-18* and *daf-16* RNAi, suggesting that PDP-1 positively modulates DAF-16 transcriptional activity. To further validate these results, we used quantitative real-time PCR (Q-PCR) to look at the expression levels of well-known DAF-16 target genes [52] in *daf-2(e1370)*, *pdp-1(tm3734)*; *daf-2(e1370)* and *daf-16(mgDf50)*; *daf-2(e1370)* worms (Figure 3C). Notably, the expression of *sod-3*, *sod-5* and *hsp-12.6* was significantly reduced in *pdp-1(tm3734)*; *daf-2(e1370)* mutants relative to *daf-2(e1370)*. Therefore PDP-1 positively regulates a subset of DAF-16 targets.

### PDP-1 acts in the DAF-7/TGF-β signaling pathway

Thus far our data indicates that PDP-1 regulates multiple outputs of IIS as well as DAF-16 activity. Using dauer formation as the readout, we performed genetic epistasis experiments to identify the substrate of PDP-1. We first tested whether *pdp-1* acted directly through the IIS pathway by focusing on kinase mutants downstream of *daf-2* (Table 1 and Figure S9). *pdk-1(sa680)*, *daf-2(e1370)*; *akt-1(ok525)* and *daf-2(e1370)*; *akt-2(ok393)* mutants were maintained on vector, *daf-18* and *pdp-1* RNAi and dauer formation of these strains was assayed at the appropriate temperatures. Interestingly, *pdp-1* RNAi resulted in suppression of dauer formation of *pdk-1(sa680)* mutants, *daf-2(e1370)*; *akt-1(ok525)* and *daf-2(e1370)*; *akt-2(ok393)* worms (Table 1 and Figure S9). DAF-16 is downstream of the AKT kinases in the pathway, but we were unable to detect a physical interaction between PDP-1 and DAF-16 (data not shown).

**Table 1:** Genetic epistasis analysis using IIS mutants.

	% Dauers ± Std. Dev (n)		
Strain	vector RNAi	*daf-18* RNAi	*pdp-1* RNAi
*pdk-1(sa680)* a ^,^ b	85.0±4.7 (520)	-	35.3±2.5 (327)*
*daf-2(e1370)* c	8.3±8.6 (476)	0 (331)	5.5±1.0 (241)
*da f-2(e1370)*; *akt-1(ok595)* c	36.9±1.4 (390)	3.5±0.9 (265)*	16.0±0.4 (375)*
*daf-2(e1370)* d	75.6±4.8 (247)	0.3±1.0 (777)*	17.3±8.2 (597)**
*daf-2(e1370)*; *akt-2(ok393)* d	61.1±15.3 (289)	4.1±1.7 (308)**	11.5±3.6 (301)**

Assays were performed at

a 22.5°C,

c 19.2°C and

d 20°C.

b As previously reported, *pdk-1(sa680)* mutants survive poorly on *daf-18* RNAi.

*p<0.01.

**p<0.05.

We next examined a TGF-β pathway that also regulates dauer formation [19]–[21] using genetic epistasis analyses with mutants of this pathway. In these assays, TGF-β pathway mutants were maintained on vector RNAi, *pdp-1* RNAi and *daf*-3 RNAi (as a positive control; Table 2 and Figure S10). We first tested *daf-7* mutants, which contain a mutation in the gene encoding the TGF-β ligand [53]. Dauer formation of *daf-7(e1372)* mutants was suppressed on *pdp-1* RNAi similar to *daf-3* RNAi, suggesting that *pdp-1* does not function at the level of *daf-7* (Table 2 and Figure S10). Next, we tested dauer formation with mutants of the SMADS *daf-8* and *daf-14* [22]. We grew *daf-14(m77)* mutants on vector, *pdp-1* and *daf-3* RNAi. Interestingly, *pdp-1* RNAi had no effect on *daf-14* dauer formation, while *daf-3* RNAi still resulted in suppression (Table 2 and Figure S10). We next looked at dauer formation of *daf-8(m85)* mutants and again observed that *pdp-1* RNAi had no effect, while *daf-3* RNAi suppressed dauer formation (Table 2 and Figure S10). Therefore, our genetic epistasis results indicate a genetic interaction between *pdp-1* and *daf-14/daf-8*.

**Table 2:** Genetic epistasis analysis using TGF-β signaling mutants.

	% Dauers ± Std. Dev (n)		
Strain	vector RNAi	*daf-3* RNAi	*pdp-1* RNAi
*daf-7(e1372)* a	85.3±1.1 (612)	43.4±0.8 (134)*	32.2±4.9 (122)*
*daf-14(m77)* b	81.7±5.6 (543)	18.1±8.9 (441)**	88.7±1.3 (535)
*daf-8(m85)* a	32.0±9.7 (392)	2.3±1.8 (396)**	34.6±9.1 (430)
*daf-2(e1370)*; *daf-3(mgDf90)* c	50.8±0.4 (302)	-	49.5±2.5 (270)

Assays were performed at

a 22.5°C,

b 20°C and

c 19.2°C.

*p<0.01.

**p<0.05.

To confirm these results, we investigated whether *pdp-1* RNAi could suppress dauer formation of *daf-2(e1370)*; *daf-3(mgDf90)* double mutants (Table 2 and Figure S10). In this strain, input from the TGF-β pathway is removed due to the *daf-3* null mutation, and dauer formation is presumably mediated through activated DAF-16 [39]. Therefore, if *pdp-1* was indeed acting in the TGF-β pathway, we would not see any effect of *pdp-1* RNAi on *daf-2(e1370)*; *daf-3(mgDf90)* double mutants. Expectedly, *pdp-1* RNAi had no effect on *daf-2(e1370)*; *daf-3(mgDf90)* double mutants (Table 2 and Figure S10). DAF-3 itself is unlikely to be a substrate for PDP-1, as similar to mammalian Co-SMADs, it lacks the SMAD phosphorylation motif [28]. Therefore, our genetic epistasis analysis supports a model whereby *pdp-1* acts in the DAF-7 TGF-β pathway at the level of *daf-8* and *daf-14*.

### TGF-β signaling can modulate the IIS pathway

How does a phosphatase in the TGF-β signaling pathway have such robust effects on the outputs of the IIS pathway and DAF-16? A number of studies have previously identified roles for the TGF-β pathway in lifespan and fat storage [7], [54], [55]. However, genetic epistasis analysis on dauer formation placed DAF-7 TGF-β signaling and IIS as two parallel pathways where components of one pathway did not affect the other [14], [56], [57]. Yet in our studies, PDP-1 was able to regulate multiple outputs of IIS. Therefore, we decided to further investigate the potential crosstalk between the IIS and TGF-β signaling pathways. First, we focused on DAF-3 and DAF-5, which are positive regulators of dauer formation similar to PDP-1, and asked whether mutations in *daf-3* or *daf-5* could also affect phenotypes of the IIS pathway [14], [28], [29].

**Figure 4:**
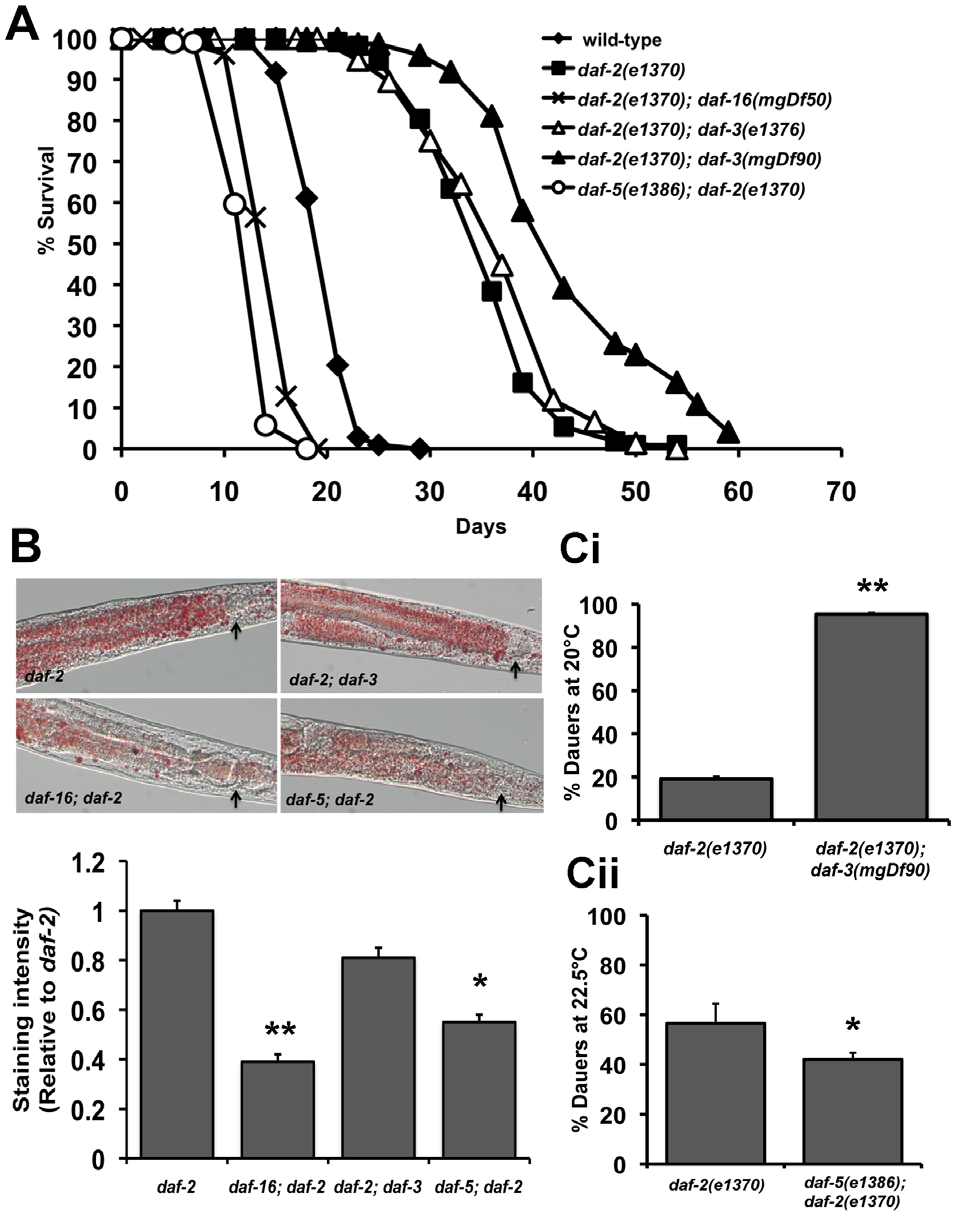
Crosstalk between IIS and TGF-β signaling pathways. Data shown are from one representative experiment. For the dauer assays, error bars indicate the standard deviation among the different plates within one experiment. (A) Lifespan graph showing the opposite effects of *daf-3* and *daf-5* mutations in a *daf-2(e1370)* background. Mean survival of wild-type worms is 20.1±0.2 days (n = 108), *daf-2(e1370)* worms is 35.6±0.5 days (n = 111), *daf-16(e1370)*; *daf-2(e1370)* worms is 14.9±0.6 days (n = 74) p<0.0001, *daf-2(e1370)*; *daf-3(e1376)* worms is 37.2±0.8 days (n = 76) p<0.02 , *daf-2(e1370)*; *daf-3(mgDf90)* is 40.5±0.7 days (n = 57) p<0.0001 and *daf-5(e1386)*; *daf-2(e1370)* worms is 13.0±0.2 days (n = 104), p<0.0001). (B) Top panel: Oil Red O staining showing the modulation of fat stores in *daf-2(e1370)* by mutations in *daf-16*, *daf-3* and *daf-5*. Arrows indicate the lower bulb of the pharynx. Lower panel: Quantification of Oil Red O staining shows a significant reduction in *daf-2(e1370)* fat storage by a mutation in *daf-16* (p<0.0001) and *daf-5* (p<0.0001). (C) *daf-2(e1370)* dauer formation is enhanced or decreased by mutations in *daf-3* and *daf-5*. i) Dauer formation of *daf-2(e1370)* is 19.2±0.7% (n = 1062) and *daf-2(e1370)*; *daf-3(mgDf90)* is 95.3±0.3% (n = 393), p<0.0001. (ii) Dauer formation of *daf-2(e1370)* is 57.1±9.8% (n = 176) and *daf-5(e1386)*; *daf-2(e1370)* is 43.8±5.4% (n = 396), p<0.02.

We tested lifespan, fat storage, dauer formation and stress resistance of TGF-β pathway mutants in a wild-type as well as *daf-2(e1370)* background. (Figure 4A–4C, Figure S11, 12, S13 and Table S1). As previously reported, the lifespan of *daf-3* and *daf-5* single mutants is slightly shorter than wild-type worms (Table S1) [55]. In our hands, mutations in the upstream components of the TGF-β pathway such as *daf-7* and *daf-14* enhance dauer formation but do not significantly extend lifespan (Table S1 and Figure S4). Intriguingly, mutations in *daf-3* and *daf-5* have opposite effects on *daf-2(e1370)* phenotypes. When compared to the *daf-2(e1370)* parental strain, *daf-2(e1370)*; *daf-3(mgDf90)* mutants lived significantly longer. This was also observed in *daf-2(e1370)*; *daf-3(e1376)* worms, which is a weaker allele of *daf-3*. In contrast, *daf-5(e1386)*; *daf-2(e1370)* double mutants live much shorter than *daf-2(e1370)* worms (Figure 4A, Figure S13 and Table S1). A mutation in *daf-5* also decreased the increased lifespan of *age-1(hx546)* worms, with *age-1(hx546)*; *daf-5(e1385)* double mutants living significantly shorter than the parental strain (Figure S13). Importantly, for *daf-2* worms, the effect of a *daf-3* null mutation on lifespan was more pronounced at 20°C where signaling through the IIS pathway is further reduced. Therefore, under low IIS conditions, DAF-3 as well as DAF-5 can modulate longevity.

We next tested the role of DAF-3 and DAF-5 on fat storage, dauer formation and stress resistance. Oil Red O staining for fat storage showed comparable levels between *daf-2(e1370)* and *daf-2(e1370)*; *daf-3(mgDf90)* worms, but markedly lesser amounts of fat in *daf-5(e1386)*; *daf-2(e1370)* worms (Figure 4B top and bottom panel and Figure S12). Similarly, *age-1(hx546)*; *daf-5(e1385)* had less fat than *age-1(hx546)* worms (Figure S12). Both *daf-3* and *daf-5* single mutants have slightly reduced levels of fat when compared to wild-type worms (Figure S12).

A similar trend was seen with our data for dauer formation. *daf-2(e1370)*; *daf-3(mgDf90)* worms show significant enhancement of *daf-2(e1370)* dauer formation across several temperatures tested, whereas a *daf-5* mutation or *daf-5* RNAi results in reduced *daf-2(e1370)* dauer formation (Figure 4Ci, Figure 4Cii and Figure S11). In addition, *daf-5(e1386)*; *daf-2(e1370)* worms fail to completely arrest at the restrictive temperature of 25°C (data not shown). A mutation in *daf-5* also significantly reduces thermotolerance of *daf-2(e1370)* worms at 37°C (Figure S13). Taken together, similar to PDP-1, DAF-3 and DAF-5 modulate multiple outputs of the IIS pathway. Unexpectedly, we find that while DAF-3 promotes dauer formation under conditions of reduced TGF-β signaling, it negatively regulates dauer formation and longevity under conditions of reduced IIS.

To further explore the crosstalk between both pathways, we next asked whether DAF-18 and DAF-16, which are components of the IIS pathway, affect TGF-β pathway signaling. For this, we assayed dauer formation of TGF-β pathway mutants on *daf-18* and *daf-16* RNAi (Table 3 and Figure S10). Interestingly, dauer formation of *daf-7(e1372)*, *daf-14(m77)* and *daf-8(m8 5)* worms was robustly suppressed by *daf-16* RNAi. We observed similar results for dauer formation *daf-7(e1372)* and *daf-14(m77)* mutants on *daf-18* RNAi. However, in the case of *daf-8(m85)* mutants, *daf-18* RNAi had no effect on dauer formation of (Figure S10), suggesting a complex crosstalk between both pathways. The enhanced dauer formation of *daf-2(e1370)*; *daf-3(mgDf90)* is suppressed by both *daf-18* and *daf-16* RNAi but not *pdp-1* RNAi (Table 3 and Figure S10). Therefore, we not only observe DAF-3 and DAF-5 affecting various phenotypes of the IIS pathway, but also the converse, where DAF-16 and DAF-18 robustly regulates TGF-β dauer formation. These results unravel a more complex interaction between the two pathways, where DAF-16 is likely to be the major downstream effector regulating longevity, dauer formation and other physiological outputs.

**Table 3:** Dauer formation of TGF-β signaling mutants is regulated by DAF-18 and DAF-16.

	% Dauers ± Std. Dev (n)			
Strain	vector RNAi	*daf-3* RNAi	*daf-18* RNAi	*daf-16* RNAi
*daf-7(e1372)* a	93.4±3.6 (113)	52.3±1.0 (683)**	44.5±1.4 (79)*	43.1±1.9 (72)**
*daf-14(m77)* b	73.1±9.4 (361)	51.5±4.9 (524)#	41.5±1.4 (500)*	23.2±5.0 (152)#
*daf-8(m85)* c	99.3±0.5 (441)	75.7±0.1 (580)**	nt	4.3±4.3 (270)*
*daf-2(e1370)*; *daf-3(mgDf90)* d	47.4±2.0 (364)	-	5.9±1.0 (314)**	0 (240)

Assays were performed at

a 22.5°C,

b 20°C,

c 25°C and

d 19.2°C respectively.

nt – not tested at this temperature. Assays with *daf-18* RNAi are in the supplementary data.

*p<0.01.

**p<0.005.

**#:** p<0.05.

### Insulins are a possible connection between TGF-β signaling and IIS

**Figure 5:**
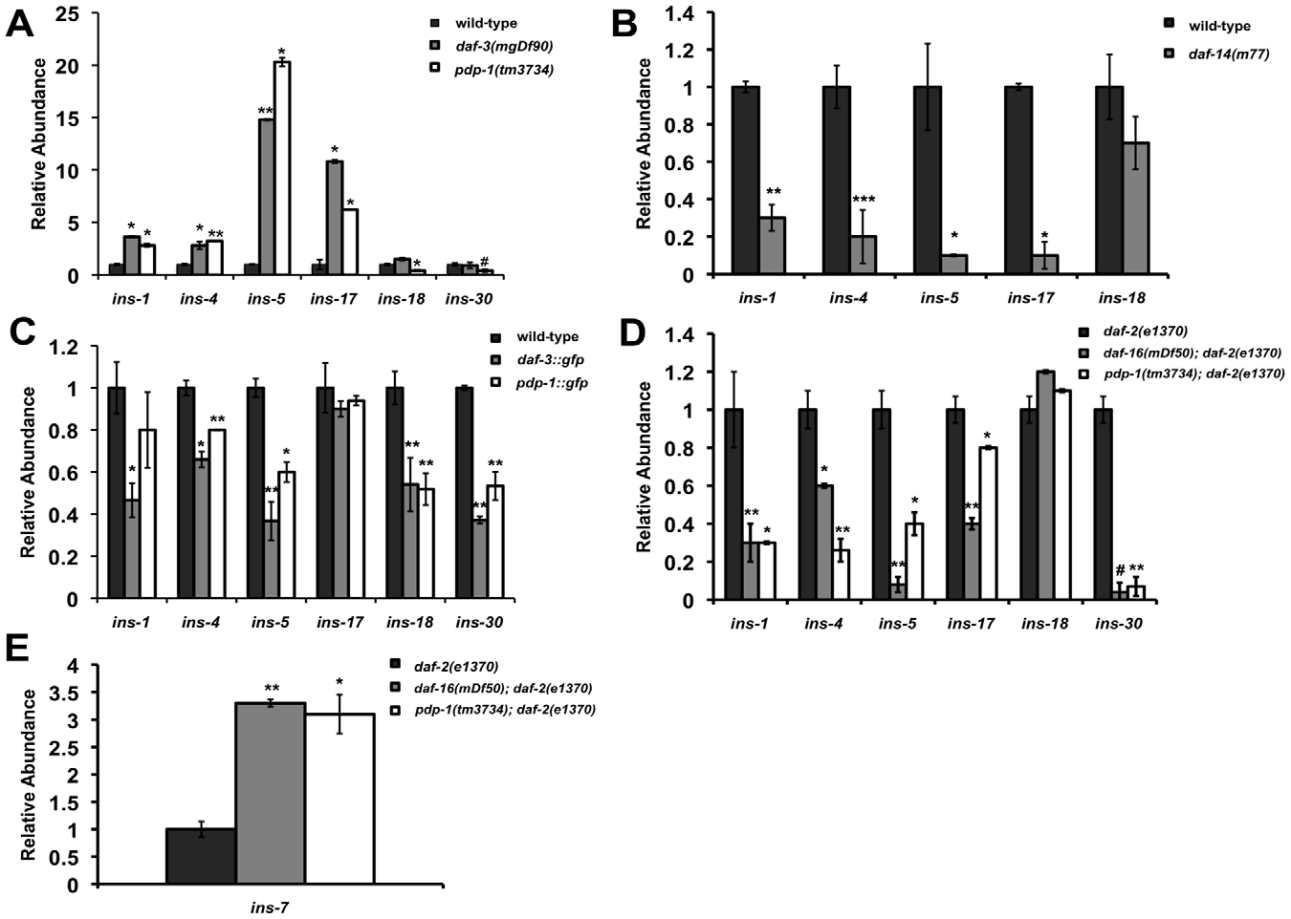
PDP-1 modulates the expression of insulin genes that possibly feed into the IIS pathway. Data are representative of one experiment. Error bars represent standard error of the mean within triplicates. All experiments were performed at least twice. (A) The expression of several insulins is elevated in both *pdp-1(tm3734)* and *daf-3(mgDf90)* mutants. * p<0.05, ** p<0.007, ^#^ a significant reduction in *ins-30* levels is observed in *pdp-1(tm3734)* worms this set but not in others, p<0.03. (B) The same insulins show decreased expression on *daf-14(m77)* mutants. *p<0.008, **p<0.005, ***p<0.0001. (C) In contrast to the mutants, *daf-3::gfp* and *pdp-1::gfp* worms show reduced levels of the insulins tested. *p<0.05, **p<0.005. (D) The trend in Insulin levels are similar between *pdp-1(tm3734)*; *daf-2(e1370)* and *daf-16(mgDf50)*; *daf-2(e1370)* double mutants compared to the *daf-2(e1370)* parental strain. *p<0.03, **p<0.01, ^#^ a significant reduction in *ins-18* levels is observed in *daf-16(mgDf50*; *daf-2(e1370)* mutants this set but not in others, p<0.001. (E) *ins-7* levels are drastically elevated in *daf-16(mgDf50)*; *daf-2(e1370)* and *pdp-1(tm3734)*; *daf-2(e1370)* worms as compared *daf-2(e1370)* worms. *p<0.01, **p<0.004.

How can these two pathways, once considered to be parallel to each other, be mechanistically linked? Thus far our data suggests that PDP-1, a component of the TGF-β pathway can modulate multiple phenotypes of IIS by positively regulating DAF-16. In addition, we observe extensive crosstalk between the two pathways at multiple levels. A feed-forward model that has been proposed to connect TGF-β signaling to the IIS pathway suggests insulins as a possible link [55], [58]. The *C. elegans* genome encodes 40 insulin genes [59], [60] (WormBase 215: www.wormbase.org). Studies using mutants and RNAi have characterized some of the insulins as agonists or antagonists of the IIS pathway [13], [59]–[61]. Importantly, microarray studies have identified several insulin genes that are regulated by TGF-β signaling, including *ins-1*, *ins-4*, *ins-5*, *ins-6*, *ins-7*, *ins-17*, *ins-18*, *ins-30*, *ins-33*, *ins-35* and *daf-28* [55], [57]. We tested changes in the levels of these insulins using Q-PCR in TGF-β pathway mutants such as *daf-3(mgDf90)*, *daf-14(m77)* as well as *pdp-1(tm3734)* and compared them to wild-type worms (Figure 5A–5C, Figure S14, Tables S2 and S3). Interestingly, both *pdp-1(tm3734)* and *daf-3(mgDf90)* showed elevated levels of several insulins as compared to wild-type worms (Figure 5A and Figure S14). In contrast, expression of these insulins was markedly reduced in *daf-14(m77)* mutants (Figure 5B and Figure S14). We next looked at the effects of overexpressing DAF-3 and PDP-1 on insulin gene expression (Figure 5C and Figure S14). The levels of several insulins are markedly reduced in *daf-3::gfp* and *pdp-1::gfp* animals when compared to wild-type worms. Therefore, dosage modulation of DAF-3 and PDP-1 modulates insulin gene expression. INS-4, for example, has been reported as a positive regulator TGF-β pathway and a suppressor of dauer formation of *daf-7* and *daf-8* mutants [62]. *ins-4* transcript levels were elevated in *pdp-1* and *daf-3* mutants but reduced in *daf-14*.

To investigate insulin gene expression regulated by DAF-16, we tested *daf-2(e1370)*, *pdp-1(tm3734)*; *daf-2(e1370)* and *daf-16(mgDf50)*; *daf-2(e1370)* mutants. Several insulins were changed relative to *daf-2(e1370)* worms, with the trend between *pdp-1(tm3734)*; *daf-2(e1370)* and *daf-16(mgDf50)*; *daf-2(e1370)* being quite similar (Figure 5D and Figure S14). Interestingly, *ins-7* levels were elevated both double mutants (Figure 5E and Figure S14). Previous studies have shown *ins-7* to be an agonist of the IIS pathway as well as a DAF-16 target gene [13], [63]. In contrast, *ins-1* levels were drastically reduced, and INS-1 has been characterized as a potential antagonist of IIS [59]. We did not observe a significant change in *ins-18*, another potential DAF-16 target [13]. We also did not detect any appreciable differences in insulin gene expression in *daf-16(mgDf50)* single mutants (Figure S14). In addition, we were unable to detect *ins-33* and *ins-35* transcripts in all the strains tested, and the trend observed with *daf-28* was inconclusive (Table S2 and S3). Taken together, our results suggest the possibility that insulins downstream of TGF-β signaling mediate at least part of the cross talk between the two pathways. Therefore, PDP-1 would modulate to regulate expression of several insulins that can potentially feed into or antagonize the IIS pathway to regulate DAF-16 and its associated phenotypes.

## Discussion

We identified *pdp-1* from a RNAi screen for serine/threonine phosphatases that modulate *daf-2* dauer formation. *C. elegans* PDP-1 is homologous to mammalian pyruvate dehydrogenase phophatase (PDP), a metabolic enzyme that is a positive regulator of the pyruvate dehydrogenase enzyme complex (PDHc). Remarkably, other components of the PDHc in *C. elegans* do not affect *daf-2* dauer formation. Microarray and SAGE studies on dauers have indicated that genes involved in anaerobic metabolism are upregulated while genes involved in the TCA cycle and mitochondrial oxidative phosphorylation are downregulated, suggesting that PDHc activity may not be critical for dauer diapause [64]–[66]. Further, annotations indicate that the *C. elegans* genome encodes approximately 60 serine/threonine phosphatases, in contrast to the 400 plus protein kinases, suggesting that phosphatases are likely to have a number of cellular substrates [39], [67]. We find that PDP-1 also regulates longevity, fat storage and stress resistance in addition to dauer formation. Interestingly, these phenotypes are more severe in mutants such as *daf-2* and *age-1*, where IIS is reduced. Further, PDP-1 positively regulates DAF-16 activity. We reason that PDP-1 function is critical under conditions of stress or low food availability, when DAF-16 activation is required [39].

Intriguingly, genetic epistasis analyses place PDP-1 in the DAF-7/TGF-β pathway, at the level of the R-SMAD proteins DAF-14 and DAF-8. A recent functional RNAi screen for serine/threonine phosphatases that modulate BMP signaling identified PDP as a SMAD1 phosphatase in *Drosophila* S2 cells and mammalian 293T cells [68]. Our study complements these findings and reveals a molecular conservation in the role of PDP-1 in regulating TGF-β signaling. Early genetic epistasis studies had suggested that TGF-β signaling and IIS pathways are parallel signaling pathways that modulate dauer diapause [31]. Importantly, in these studies, the conclusion was that both these pathways acted independently, and it was the IIS pathway that regulated longevity and stress resistance [31], [32].

**Figure 6:**
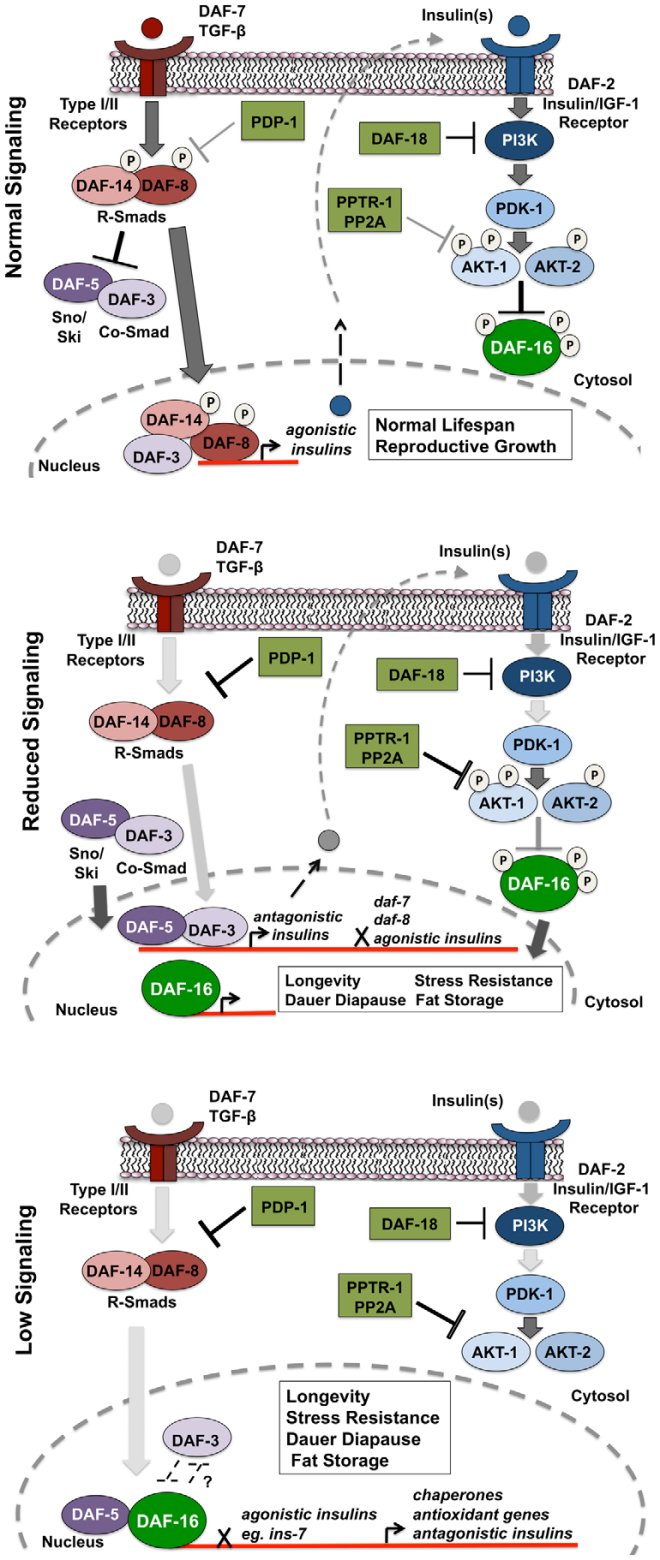
PDP-1 links TGF-β signaling to the IIS pathway and DAF-16. Top panel: Under favorable environmental conditions, signaling through the TGF-β pathway activates the R-SMAD proteins DAF-8 and DAF-14, which regulate insulin gene expression while antagonizing DAF-3 and DAF-5 function. These insulins may act as agonists and activate IIS, thereby promoting phosphorylation and suppression of DAF-16 activity. In this feed-forward model, the worm undergoes reproductive growth and has a normal life span. Middle panel: PDP-1 negatively regulates TGF-β signaling through dephosphorylation of DAF-8 and DAF-14. Under these conditions, DAF-3 and DAF-5 repress the transcription of agonistic insulins as well as expression of the *daf-7* TGF-β ligand and *daf-8*, leading to further downregulation of the TGF-β pathway. Alternatively, DAF-3 and DAF-5 may promote transcription of potential antagonistic insulins. This results in reduced signaling through the IIS pathway, enhancing DAF-16 nuclear localization. Lower panel: Under low IIS conditions, DAF-16 localization is predominantly nuclear, where it regulates the transcription of hundreds of target genes that act in combination to regulate longevity, stress resistance, dauer formation and the response to stress. Paradoxically, under low IIS conditions, DAF-3 and DAF-5 play opposite roles. DAF-5 is likely to synergize with DAF-16 and modulate the activity of its target genes. DAF-3 acts to antagonize DAF-16, either directly or through suppression of DAF-16 target genes. Therefore, the role of DAF-3 in modulating IIS depends upon the level of signaling through the pathway.

However, the effect of PDP-1 on DAF-16 activity led us to re-investigate the interaction between the IIS and TGF-β signaling. Previous studies have shown that DAF-3 and DAF-5 are negatively regulated by TGF-β signaling, and function similarly as repressors of gene expression to ultimately promote dauer formation [28],[29],[69],[70]. We find that under conditions of reduced IIS, DAF-3 and DAF-5 affect various outputs of the IIS pathway in opposite ways. DAF-3 in particular regulates IIS depending upon the level of signaling through the pathway (Figure 6). In our hands, mutants of the TGF-β signaling pathway do not exhibit a pronounced increase in lifespan. However, components of this pathway are important for the long lifespan of mutants in the IIS pathway, as well as other phenotypes such as dauer formation, fat storage and stress resistance. Our epistasis studies reveal that *daf-18* and *daf-16* RNAi can strongly suppress dauer and fat storage of TGF-β pathway mutants. Together, these results point to a feed-forward model where signals through the TGF-β pathway are relayed to modulate activity of the IIS pathway as well as DAF-16. Indeed, recent studies have suggested that TGF-β pathway regulates the expression of insulins, leading to a feed-forward model, where signals from the TGF-β pathway are relayed to modulate activity of the IIS pathway as well as DAF-16 [55], [58].

In support of this model, we find TGF-β signaling regulates the expression of several insulin genes with DAF-3 and PDP-1 negatively modulating insulin gene expression. This is in agreement with previous studies that identify DAF-3 as a repressor of gene expression [69], [70]. The expression of several insulins is also modulated by DAF-16, with *pdp-1(tm3734)*; *daf-2(e1370)* and *daf-16(mgDf50)*; *daf-2(e1370)* worms showing similar trends in insulin levels. Therefore, in the absence of PDP-1, increased levels of agonists or reduced levels of antagonists hyperactivate the DAF-2 pathway to negatively regulate DAF-16, thereby affecting the enhanced lifespan, stress resistance, dauer formation and fat storage of *daf-2* mutants.

Our results suggest a model where under favorable growth conditions, signals through the TGF-β pathway activate the SMAD transcriptional complex to regulate the expression of insulins that activate the IIS pathway to phosphorylate and inhibit DAF-16 activity, thereby promoting growth, reproduction and normal lifespan (Figure 6, top panel). However, when food is limiting or under harsh survival conditions, TGF-β signaling is downregulated by PDP-1 to activate DAF-3 and DAF-5, to regulate the repression of insulin genes that may feed into the IIS pathway (Figure 6, middle panel). DAF-3 has also been reported to negatively regulate *daf-7* and *daf-8* gene expression in a feedback loop [24]. We find that *pdp-1* expression is elevated in *daf-3(mgDf90)* mutants, suggesting a similar feedback regulation (Figure S15). Repression of TGF-β and insulin gene expression by DAF-3 results in a reduction in signaling through the IIS pathway, and promotes DAF-16 nuclear localization. DAF-16 then regulates the transcription of hundreds of target genes that ultimately modulate longevity, stress resistance, dauer formation and fat storage. Under low TGF-β signaling and IIS conditions, DAF-3 and DAF-5 regulate these outputs in an opposite manner, with DAF-5 synergizing and DAF-3 antagonizing DAF-16 function (Figure 6 lower panel). With our Q-PCR data, we found that PDP-1 affected only a subset of the DAF-16 target genes tested. These could represent genes that are regulated by DAF-16 and SMAD proteins. SMAD proteins have low affinity for binding DNA, and the orchestration of cellular signals into defined outputs requires their association with additional co-factors [71]. Mammalian SMAD proteins can bind several co-activators and co-repressor proteins to modulate gene transcription [23]. Specifically, a synergy between mammalian FOXO (FOXO1, FOXO3a and FOXO4) and SMAD2/3 was identified for the regulation of several genes involved in cell cycle regulation and the response to stress [72]. Importantly, these interactions required the function of the co-SMAD protein SMAD-4, which is homologous to DAF-3 [72]. Therefore, DAF-3 and DAF-5 could also directly modulate the IIS pathway at the transcriptional level.

A clear interpretation of our results is complicated by three main factors. First, the sheer number of insulins in the worm makes it difficult to assess whether they are functionally distinct. Secondly, the role of temperature in modulating the readouts of the pathway has not been closely explored. For example, we observe the effects of *pdp-1* RNAi on *daf-2* lifespan at 15°C but the effect decreases at a higher temperature, as the pathway gets more inactive. It is therefore likely that a certain level of signaling through the pathway is required to activate and target PDP-1 to its substrate(s). At higher temperatures such as 20°C or 25°C, there may be extremely low levels of phosphorylated substrate available for PDP-1. Similarly, the effect of a *daf-3* null mutation on *daf-2* phenotypes is more pronounced at higher temperatures but not at 15°C. Third, the lack of null alleles may provide an incomplete picture of the phenotypes observed. For example, previous studies using non-null alleles of *daf-16* only partially suppressed dauer formation of TGF-β pathway mutants and therefore DAF-16 was thought to only affect the IIS pathway [31]. Therefore, temperature, level of signaling and the kind of mutants used (null versus weak) are important additional inputs that need to be considered to better understand the crosstalk between the IIS and the TGF-β pathways.

In conclusion, our studies show that PDP-1 acts through the TGF-β pathway to negatively regulate IIS and promote DAF-16 activity. PDP-1 may mediate this function in part by negatively regulating TGF-β signaling to repress expression of several insulins that feed into the IIS pathway. In humans, dysregulation of TGF-β signaling and the insulin/IGF-1 signaling axis have been implicated in the onset of age-associated diseases such as Type 2 Diabetes and cancer [73]–[77]. Future studies exploring the interactions between these two pathways as well as the factors that modulate these interactions may ultimately provide a better understanding of the pathophysiology of these diseases.

## Materials and Methods

### Strains

All strains were maintained at 15°C using standard *C. elegans* techniques [78]. For all RNAi assays, worms were maintained on the RNAi bacteria for two generations except for the assays on the PDHc RNAi. Strains used in this manuscript are listed in Table S4.

### RNAi–based assays

RNAi plates were prepared as previously described [39]. All RNAi clones were sequenced and verified before any assays were carried out. L4 worms were picked onto fresh RNAi plates and maintained for two generations prior to the assay, with the exception PDHc RNAi plates. Worms exhibit lethality when maintained on the following RNAi clones: T05H10.6 (E1α), C04C3.3 (E1β), F23B12.5 (E2), or LLC1.3 (E3) [79]. To circumvent this problem, strains were maintained on vector RNAi for two generations and transferred to E1α, E1β, E2 or E3 plates prior to the assay.

### Strain construction

For the *pdp-1(tm3734)*;*daf-2(e1370)* double mutant, *daf-2(e1370)* males were mated to *pdp-1(tm3734)* hermaphrodites at 15°C. A total of 30 F1 progeny were picked onto individual plates and allowed to have progeny at 25°C. From the F2 progeny on each plate, dauers were selected and transferred to fresh plates and incubated for an additional 24 hours at 25°C. The next day, the dauers were allowed to recover at 15°C until they reached adulthood. Subsequently, adult worms were picked onto individual plates and transferred to 25°C and allowed to have progeny. Among the F3 progeny, we observed that some plates had 100% dauers at 25°C, while worms in some of the plates exhibited a developmental delay and could not form complete dauers even after 5–6 days at 25°C. Worms from both sets of plates were recovered, picked to individual plates and allowed to self at 15°C. Parents were then tested for *pdp-1(tm3734)* deletion by PCR. As anticipated, the *pdp-1(tm3734)*;*daf-2(e1370)* double mutants are unable to form 100% dauers at 25°C.

The *daf-2(e1370)*;*pdp-1::gfp* strain was made by crossing *daf-2(e1370)* males to *pdp-1::gfp* hermaphrodites at 15°C. About 30 F1 animals were transferred to individual plates and allowed to have progeny at 25°C. From the progeny, F2 dauers were selected from each plate and allowed to recover at 15°C. The recovered adult worms were then checked for the presence of GFP, and GFP-positive worms were transferred to individual plates and incubated at 25°C. Plates where 100% of the progeny were dauers and GFP positive were selected and established as the strain for the assays.

### Dauer assays

Strains were maintained on RNAi plates for two generations or regular OP50 plates at 15°C. Dauer assays were performed by picking approximately 100 eggs onto 2 fresh plates and incubated at the appropriate temperature. The *pdk-1(sa680)*, *daf-7(e1372)* and *daf-14(m77)* worms have a strong Egl phenotype. For dauer assays on these strains, gravid adult worms growing on the RNAi plates were washed off the plate with sterile PBS onto a 1.5 mL eppendorf tube. After 2 washes at 2000 g for 30 seconds, the adults were vortexed for 5 mins in 5 mL of 1 N sodium hydroxide and 3% sodium hypochlorite (final concentration). The samples were then washed twice with sterile PBS and eggs were aspirated with a glass pipette onto fresh RNAi plates. For all dauer assays, plates were scored for the presence of dauers or non-dauers after 3.5–5.5 days, depending upon the strain. Dauer assays were performed at the temperature indicated. Significance was determined by Student's t-test.

### Lifespan assays

Strains were maintained at 15°C and synchronized by picking eggs onto fresh RNAi or OP50 plates. Approximately 60 young adult worms were transferred per plate to a total of three fresh RNAi or regular OP-50 plates containing 5-fluorodeoxyuridine (FUDR) at final concentration of 0.1 mg/mL [80]. All RNAi-based lifespan assays were carried out at 15°C. Lifespans on OP50 plates were performed at the temperature indicated. Survival was scored by tapping with a platinum wire every 2–3 days. Worms that died from vulval bursting were censored from the analysis. Statistical analyses for survival were conducted using the standard chi-squared-based log rank test.

### Heat stress assay

Strains were maintained on RNAi or regular OP50 bacteria at 15°C, as described above. From these plates, approximately 30 young adult worms were picked onto fresh RNAi or regular plates and upshifted to 20°C for 6 hrs. The plates were then transferred to 37°C and heat stress-induced mortality was determined every few hours till all the animals died. Statistical analyses for survival were conducted using the standard chi-squared-based log rank test.

### Fat staining

Strains maintained RNAi or on regular OP50 plates were synchronized by picking eggs on to fresh plates and grown synchronously at 15°C. The plates were then upshifted to 20°C for 8 hours, at the L2 stage to get L3 worms and at the L4 stage to get young adult worms. Worms were then washed off the plates into microcentrifuge tubes and incubated in 1× PBS buffer for 20 minutes on a shaker at RT. After 2 washes at 3000 rpm for 30 seconds with 1× PBS, the strains were fixed according to the type of staining performed. Oil Red O and Sudan black staining was performed as previously described [39], [45], [81], [82]. After incubation overnight at RT, worms were mounted on slides and visualized using the Zeiss Axioscope 2+ microscope.

### Quantification of fat staining

For Sudan Black Staining, we used Image J software to measure the average pixel intensity for a 84-pixel radius below the pharynx of each animal in the anterior intestine area. Next, an 84-pixel radius of the background was measured, and subtracted from the values obtained for the staining. At least 10 animals were measured for each RNAi clone. Significance was determined by Student's t-test.

For Oil Red O Staining, Image J was used to separate out each color image into its RGB channel components. As previously described [45], Oil Red O absorbs light at 510 nm and therefore, the green channel was used for further analysis. We measured the average pixel intensity for a 84-pixel radius below the pharynx of each animal in the anterior pharynx area. We next measured a 84-pixel radius of the background, which was later subtracted from the values obtained from the staining. At least 10 animals was measured for each RNAi clone. Significance was determined by Student's t-test.

### DAF-16::GFP localization assay

DAF-16 localization assays were performed as previously described [39], [52]. *daf-2(e1370)*; *daf-16::gfp* worms were maintained on RNAi plates at 15°C similar to the dauer assays. Approximately 30 L4 worms were transferred to fresh RNAi bacteria and the plates were shifted to 20°C for 1 hr. The worms were visualized under a fluorescence microscope (Zeiss Axioscope 2+ microscope). Worms were classified into four categories based on the extent of DAF-16::GFP nuclear-cytoplasmic distribution: completely cytosolic, more cytosolic than nuclear in most tissues, more nuclear than cytosolic in most tissues and completely nuclear.

### *Psod-3::gfp* expression

Quantification of *Psod-3::gfp* was performed as previously described [39]. *daf-2(e1370)*;*sod-3::gfp* worms were grown at 15°C on RNAi as described above. Approximately 30 L4 animals were transferred to fresh RNAi bacteria and shifted to 25°C for 1 hr. The expression of *sod-3::gfp* was visualized using Zeiss Axioscope 2+ microscope. GFP expression was categorized as follows:

High: GFP expression seen throughout the worm

Medium: Weak expression detected in the body of the worm along with the head and the tail

Low: Low GFP expression only detected in the head and tail

### Transgenic worms

Promoter and ORF entry clones of *pdp-1* obtained from the promoterome and ORFeome were combined using multisite Gateway cloning (Invitrogen) into the pDEST-DD03 or the R4-R2 GFP destination vectors to create the *Ppdp-1::gfp* or *Ppdp-1::pdp-1^ORF^::gfp* constructs [83], [84]. All constructs contain the *unc-119* minigene. The vectors were verified by sequencing as well as restriction digestion. Transgenic worms were generated by ballistic transformation into *unc-119(ed3)* mutant worms as previously reported (Biorad, USA) [83]. Integrated lines that were obtained were used for further analyses. For the *pdp-1::gfp* translational fusion strain, additional lines were generated by integration of extrachromosomal array lines by UV irradiation as previously described [85]. All translational fusion lines were backcrossed 4× to wild-type prior to analysis.

### RT-PCR experiments

For all RT-PCR experiments, strains were maintained at 15°C. Eggs were obtained from gravid adult worms by hypochlorite treatment described earlier. The eggs were seeded onto large plates maintained at 15°C until the worms entered the L4 stage. The plates were then upshifted to 20°C for 8 hours until they became young adults. Worms were then collected with sterile 1×PBS and washed twice at 2000 g for 30 seconds. The supernatant was removed, and 0.5 mL of AE buffer (50 mM acetic acid, 10 mM EDTA), 0.1 mL of 10% SDS, and 0.5 mL of phenol was added to the worm pellet and the mixture was vortexed vigorously for 1 min, followed by incubation at 65°C for 4 min. Total RNA was purified by phenol:chloroform extraction and ethanol precipitation. The quality of the RNA isolated was determined by checking the 28 S and 18 S RNA on an agarose gel. 2 ug of total RNA was used for making cDNA using the SuperScript cDNA synthesis kit (Invitrogen, USA). The expression of the DAF-16 target and insulin genes was checked by RT-PCR using the SYBR Green PCR Master Mix and 7000 Real-Time PCR System (Applied Biosystems, USA). The relative expression of the genes tested was compared to actin as an internal loading control. Significance was determined by Student's t-test. Primers used for the RT-PCR experiments are listed in Table S5.

### Locomotion assay

Young adult wild-type and *pdp-1(tm3734)* worms were picked onto 6 individual plates each. After 5 minutes, the worms were picked off the plate. The average distance covered was calculated by measuring the traces on the bacterial lawn using ImageJ. Significance was determined by Student's t-test.

### Brood size measurements

Wild type, *daf-2(e1370)*, *pdp-1(tm3734)* and *pdp-1(tm3734)*; *daf-2(e1370)* worms were maintained at 15°C. 5 L4 worms were picked onto individual plates and allowed to lay eggs at 22.5°C. Worms were transferred to a new plate every 12 hours. After 22.5 hours, the parental worms were picked off the plates, and the total number of eggs laid was scored. The number of progeny from these eggs was scored again after 38 hours. The % hatched eggs was calculated as a percentage of the average number of progeny over the average number of eggs laid. Significance was determined by Student's t-test.

### Software used in this study

Statistical analyses were performed using JMP and Microsoft Excel. NIH Image J was used for quantification of locomotion and fat storage.

## Supporting Information

Figure S1 Verification of RNAi knockdown by Q-PCR. Data shown are from one representative experiment. RNAi knockdown was verified in *daf-2(e1370)* worms by Q-PCR. *^a^*For this set, verification of the knockdown for *pdhk-2* was performed independently.
(0.12 MB TIF)

Figure S2 PDP-1 regulates dauer formation independent of the PDHc. Data shown are from one representative experiment. For the dauer assays, Error bars indicate the standard deviation among the different plates within one experiment. A) *pdp-1* RNAi significantly suppresses *daf-2(e1370)* dauer formation (p<0.01), similar to *daf-18* RNAi (p<0.01) while E1α RNAi has no effect. *pdhk-2* RNAi results in a slight decrease in *daf-2(e1370)* dauer formation. B) Knockdown of components of the PDHc do not affect *daf-2(e1370)* dauer formation. RNAi of both, the E1α and E1β or the E2 subunit does not suppress dauer formation like *daf-18* RNAi (p<0.01). C) A mutation in *pdp-1* suppresses *daf-2(e1370)* dauer formation, similar to the effect of *pdp-1* RNAi. (p<0.03). D) *pdp-1* RNAi significantly suppresses *daf-2(e1368)* dauer formation (p<0.002) similar to *daf-18* RNAi (p<0.007). *pdhk-2* RNAi has no effect on *daf-2(e1368)* dauer formation. E) *pdp-1* RNAi suppresses dauer formation in *daf-2(e1370)* mutants (p<0.02) in a RNAi-sensitized background, similar to *daf-18* RNAi (p<0.02).
(0.96 MB TIF)

Figure S3 Tissue Expression patterns of PDP-1, A) Expression pattern of *pdp-1* as visualized using a *Ppdp-1::gfp* transcriptional fusion strain. Di-I staining shows co-localization in amphid neurons. B) The *Ppdp-1::gfp* strain does not show complete overlap with the expression patterns of transcriptional fusion strains of the PDHc, *PE1β::gfp* and *PE2::gfp*.
(4.41 MB TIF)

Figure S4 PDP-1 regulates lifespan. Data shown are from one representative experiment. A) *pdp-1* RNAi does not significantly reduce the lifespan of wild-type worms (p<0.07). B) *pdp-1* RNAi significantly reduces *daf-2(e1370)* lifespan (p<0.0001) similar to *daf-18* RNAi (p<0.0001). C) *pdp-1* RNAi significantly reduces *age-1(hx546)* lifespan (p<0.0001) similar to *daf-18* RNAi (p<0.0001). D) Overexpression of *pdp-1* increases lifespan (p<0.0001). E) Dosage modulation of *pdp-1* can regulate *daf-2* lifespan. *pdp-1(tm3734)*; *daf-2(e1370)* worms live significantly shorter than *daf-2(e1370)* worms (p<0.0001) while *daf-2(e1370)*; *pdp-1::gfp* worms live longer (p<0.0001). F) Mutations in *daf-14* and *daf-7* do not significantly increase lifespan. *pdp-1(tm3734)* mutants live shorter than wild-type worms (p<0.005).
(0.62 MB TIF)

Figure S5 PDP-1 regulates lifespan in a DAF-16-dependent manner. A) Increased dosage of *pdp-1* extends the lifespan of wild-type worms (p<0.005) and this extension is suppressed by *daf-16* RNAi (p<0.0001). B) Increased dosage of *pdp-1* further extends *daf-2(e1370)* lifespan (p<0.0001), and this extension is completely suppressed by *daf-16* RNAi (p<0.0001).
(0.13 MB TIF)

Figure S6 PDP-1 mutants have a slow movement phenotype and reduced brood size. Data shown are from one representative experiment. Error bars indicate the standard deviation among the different plates within one experiment. A) *pdp-1(tm3734)* mutants have a slow movement phenotype when compared to wild-type worms (p<0.001). This slow movement in the *pdp-1(tm3734)* mutant can be rescued by expression of a *pdp-1::gfp* transgene (p<0.002). Lower panel: Traces of wild-type, *pdp-1(tm3734)*, *pdp-1::gfp* and *pdp-1::gfp*; *pdp-1(tm3734)* worms moving on a lawn of OP50. B) Brood size of wild-type, *daf-2(e1370)*, *pdp-1(tm3734)* and *pdp-1(tm3734)*; *daf-2(e1370)* animals as scored after 22.5 hours (total number of eggs laid) and 38 hours (total number of progeny). C) The % hatched eggs calculated from the number of progeny and number of eggs laid. *pdp-1(tm3734)* worms have fewer progeny (p<0.04) when compared to wild-type worms, however, this phenotype is far more severe in *pdp-1(tm3734)*; *daf-2(e1370)* worms (p<0.005).
(0.82 MB TIF)

Figure S7 PDP-1 regulates stress resistance and fat storage. Data shown are from one representative experiment. Arrows indicate the lower bulb of the pharynx. A) PDP-1 regulates thermotolerance. A mutation in *pdp-1* slightly reduces thermotolerance (p<0.06) of wild-type worms but significantly reduces *daf-2(e1370)* thermotolerance (p<0.05). B) Oil Red O Staining of adult worms. Top panel: Quantification of Oil Red O staining in wild-type and *pdp-1::gfp* worms. Overexpression of *pdp-1* slightly enhances fat storage (p<0.01), and this enhancement is dependent on *daf-16* RNAi (p<0.01) but not *daf-3* or E1α RNAi. Lower panel: Oil Red O Staining of young adult worms showing comparable levels of fat between wild-type and *pdp-1(tm3734)* worms, while *pdp-1::gfp* young adults show slightly enhanced fat storage. C) Oil Red O Staining of *daf-2(e1370)* and *daf-2(e1370)*; *pdp-1::gfp* worms. Top panel: Quantification of Oil Red O staining in *daf-2(e1370)* and *daf-2*; *pdp-1::gfp* worms. Similar to *daf-2(e1370)* worms, the fat storage of *daf-2(e1370)*; *pdp-1::gfp* worms is suppressed by *daf-16* RNAi (p<0.005) but not E1αRNAi. *daf-3* RNAi slightly reduces the fat of *daf-2*; *pdp-1::gfp* but not *daf-2(e1370)* worms (p<0.01). Lower panel: Representative images of Oil Red O Staining in *daf-2(e1370)* and *daf-2(e1370)*; *pdp-1::gfp* worms on *daf-16*, *daf-3* and E1α RNAi. D) Quantification of Sudan Black Staining of *daf-2(e1370)* on different RNAi clones. The increased fat storage of *daf-2(e1370)* worms is suppressed on *daf-18* (p<0.005), *daf-16* (p<0.005), *pdp-1* (p<0.007) and *daf-5* RNAi (p<0.005).
(0.36 MB TIF)

Figure S8 PDP-1 positively regulates DAF-16 nuclear localization and activity. A) Quantification of DAF-16 subcellular localization as observed in *daf-2(e1370)*; *daf-16::gfp* worms on vector, *daf-18* and *pdp-1* RNAi. B) Quantification of GFP expression in a *daf-2(e1370)*; *Psod-3::gfp* reporter strain grown on vector, *daf-18*, *pdp-1, daf-5* and *daf-3* RNAi.
(0.47 MB TIF)

Figure S9 Epistasis analyses using mutants of the IIS pathway. A) *pdp-1* RNAi significantly suppresses dauer formation of *pdk-1(sa680)* mutants (p<0.01). B) *pdp-1* RNAi suppresses dauer formation of *daf-2(e1370)*; *akt-1(ok525)* double mutants (p<0.03). C) *pdp-1* RNAi suppresses dauer formation of *daf-2(e1370)*; *akt-2(ok393)* double mutants (p<0.05).
(0.07 MB TIF)

Figure S10 Epistasis analyses using mutants of the TGF-β pathway. A) *daf-7(e1372)* dauer formation is suppressed by *pdp-1* RNAi (p<0.02), *daf-18* RNAi (p<0.005), *daf-16* RNAi (p<0.008) as well as the controls *daf-3* RNAi (p<0.02) and *daf-5* RNAi (p<0.05). B) *pdp-1* RNAi has no effect on dauer formation of *daf-14(m77)* worms (p<0.1). However, *daf-18* RNAi (p<0.05) and *daf-16* RNAi (p<0.05) result in dauer suppression. C) *pdp-1* RNAi has no effect on dauer formation of *daf-8(m85)* worms (p<0.3). Similarly *daf-18* RNAi also has no effect on dauer formation (p<0.1). D) At 25°C *daf-16* RNAi can robustly suppress dauer formation of *daf-8(m85)* worms (p<0.009), while *daf-3* RNAi only has a partial effect (p<0.05). E) Dauer formation of *daf-2(e1370)*; *daf-3(mgDf90)* is suppressed by *daf-18* RNAi (p<0.04) but not *pdp-1* RNAi (p<0.2).
(0.70 MB TIF)

Figure S11 DAF-3 and DAF-5 regulate *daf-2(e1370)* dauer formation. Data shown are from one representative experiment. Error bars indicate the standard deviation among the different plates within one experiment. A) Dauer formation of *daf-2(e1370)*; *daf-3(e1376)* double mutants is significantly enhanced over *daf-2(e1370)* worms (p<0.004). B) Dauer formation of *daf-2(e1370)*; *daf-3(mgDf90)* double mutants is significantly enhanced over *daf-2(e1370)* worms (p<0.001). C) *daf-3* (p<0.03) and *daf-5* (p<0.06) mutations enhance and reduce *daf-2* dauer formation.
(0.32 MB TIF)

Figure S12 Crosstalk between the IIS and TGF-β signaling pathways in modulation of fat storage. Data shown are from one representative experiment. Error bars indicate the standard error among the different plates within one experiment. A) Oil Red O staining of single and double mutant adult worms of the IIS and TGF-β pathways. Arrows indicate the lower bulb of the pharynx. B) The increased fat storage of *age-1(hx546)* worms is suppressed by a mutation in *daf-5*. Top panel: Oil Red O staining of *age-1(hx546)* and *age-1(hx546)*; *daf-5(e1385)* young adult worms. Arrows indicate the lower bulb of the pharynx. Lower panel: Quantification of Oil Red O staining shows significantly reduced fat in *age-1(hx546)*; *daf-5(e1385)* worms as compared to the *age-1(hx546)* parental strain (p<0.009). C) Quantification of Sudan Black staining of *daf-7(e1372)* L3 worms on different RNAi bacteria. Fat storage of *daf-7(e1372)* animals is decreased by *daf-3* (p<0.0001), *daf-16* (p<0.001), *pdp-1* (p<0.0001), *daf-18* (p<0.0001) and *daf-5* (p<0.0001) RNAi.
(0.43 MB TIF)

Figure S13 Crosstalk between the IIS and TGF-β signaling pathways in modulation of lifespan and stress resistance. Data shown for the lifespan assays are from one representative experiment. A) Lifespan of *daf-2(e1370)*; *daf-3(mgDf90)* worms is enhanced over *daf-2(e1370)* mutants (p<0.001). *pdp-1* RNAi can significantly suppress the lifespan of *daf-2(e1370)* worms (p<0.0001) but only has a partial effect on the lifespan of *daf-2(e1370)*; *daf-3(mgDf90)* worms (p<0.01). *daf-18* RNAi significantly reduces lifespan in both strains (p<0.0001). B) *age-1(hx546)*; *daf-5(e1385)* double mutants live significantly shorter than *age-1(hx546)* worms (p<0.0001). Both *pdp-1* and *daf-18* RNAi significantly reduce the lifespan of both strains (p<0.0001). C) Survival of adult worms of the IIS and TGF-β pathways after 9.5 hours at 37°C. Data shown is an average of two independent repeats, with error bars indicating the variation between two repeats.
(0.20 MB TIF)

Figure S14 Q-PCR experiments. Data shown are from one representative experiment. Error bars represent standard error of the mean within triplicates. A) The levels of several insulin genes are elevated in *daf-3(mgDf90)* and *pdp-1(tm3734)* worms. * p<0.05, **p<0.02, ^#^p<0.0009. B) The levels of the same set of insulins are markedly decreased in *daf-14(m77)* worms. *p<0.05, **p<0.0007. C) Insulins levels are significantly decreased in *daf-3::gfp* and *pdp-1::gfp* strains. *p<0.05, **p<0.01, ^#^p<0.001. D) Insulin gene regulation is under the control of the IIS pathway. Compared to *daf-2(e1370)* worms, the levels of several insulins change in *pdp-1(tm3734)*; *daf-2(e1370)* worms and *daf-16(mgDf50)*; *daf-2(e1370)* worms. *p<0.05, **p<0.0007, ^#^p<0.0005. E) *ins-7* levels are significantly increased in *daf-16(mgDf50)*; *daf-2(e1370)* and *pdp-1(tm3734)*; *daf-2(e1370)* double mutants, compared to *daf-2(e1370)*. *p<0.04, **p<0.001. F) The levels of several insulin genes are unchanged in *daf-16(mgDf50)* single mutants. *p<0.05.
(0.58 MB TIF)

Figure S15 DAF-3 regulates *pdp-1* expression. A) Q-PCR results showing elevated levels of *pdp-1* in *daf-3(mgDf90)* mutants. Also, expression is slightly increased over wild-type worms in *daf-2(e1370)* mutants but decreased in *daf-16(mgDf50)* worms as well as *daf-16(mgDf50)*; *daf-2(e1370)* worms. Data shown are from one representative experiment. Error bars represent standard error of the mean within triplicates. B) Compared to vector RNAi, GFP expression of the *Ppdp-1::gfp* transcriptional fusion strain is higher on *daf-3* RNAi, and slightly reduced on *daf-16* RNAi (100× magnification).
(0.30 MB TIF)

Table S1 Lifespans of IIS and TGF-β pathway mutants.
(0.04 MB DOC)

Table S2 List of insulins tested in this manuscript.
(0.04 MB DOC)

Table S3 Summary of trends observed in the Q-PCR Experiments.
(0.05 MB DOC)

Table S4 List of strains used in this manuscript.
(0.06 MB DOC)

Table S5 List of primers used in this manuscript.
(0.13 MB DOC)
