# Supplementary figures and images for: PDP-1 Links the TGF-β and IIS Pathways to Regulate Longevity, Development, and Metabolism
Source: PLoS Genet. 2011 Apr 21;7(4):e1001377. doi: 10.1371/journal.pgen.1001377 (PMC3080858; doi:10.1371/journal.pgen.1001377)

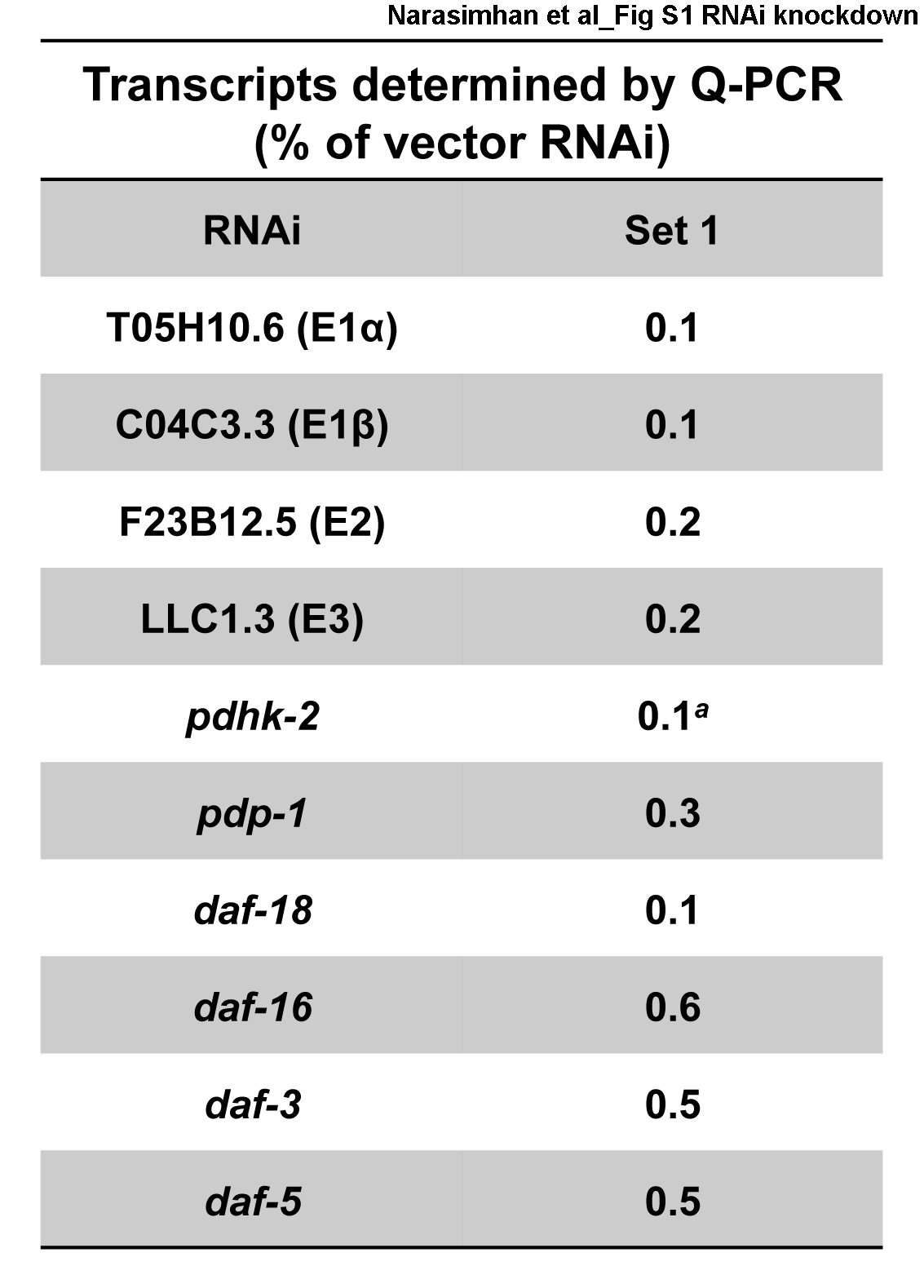

Supplement: Figure S1 — Verification of RNAi knockdown by Q-PCR. Data shown are from one representative experiment. RNAi knockdown was verified in daf-2(e1370) worms by Q-PCR. aFor this set, verification of the knockdown for pdhk-2 was performed independently. (0.12 MB TIF) [file pgen.1001377.s001.tif]

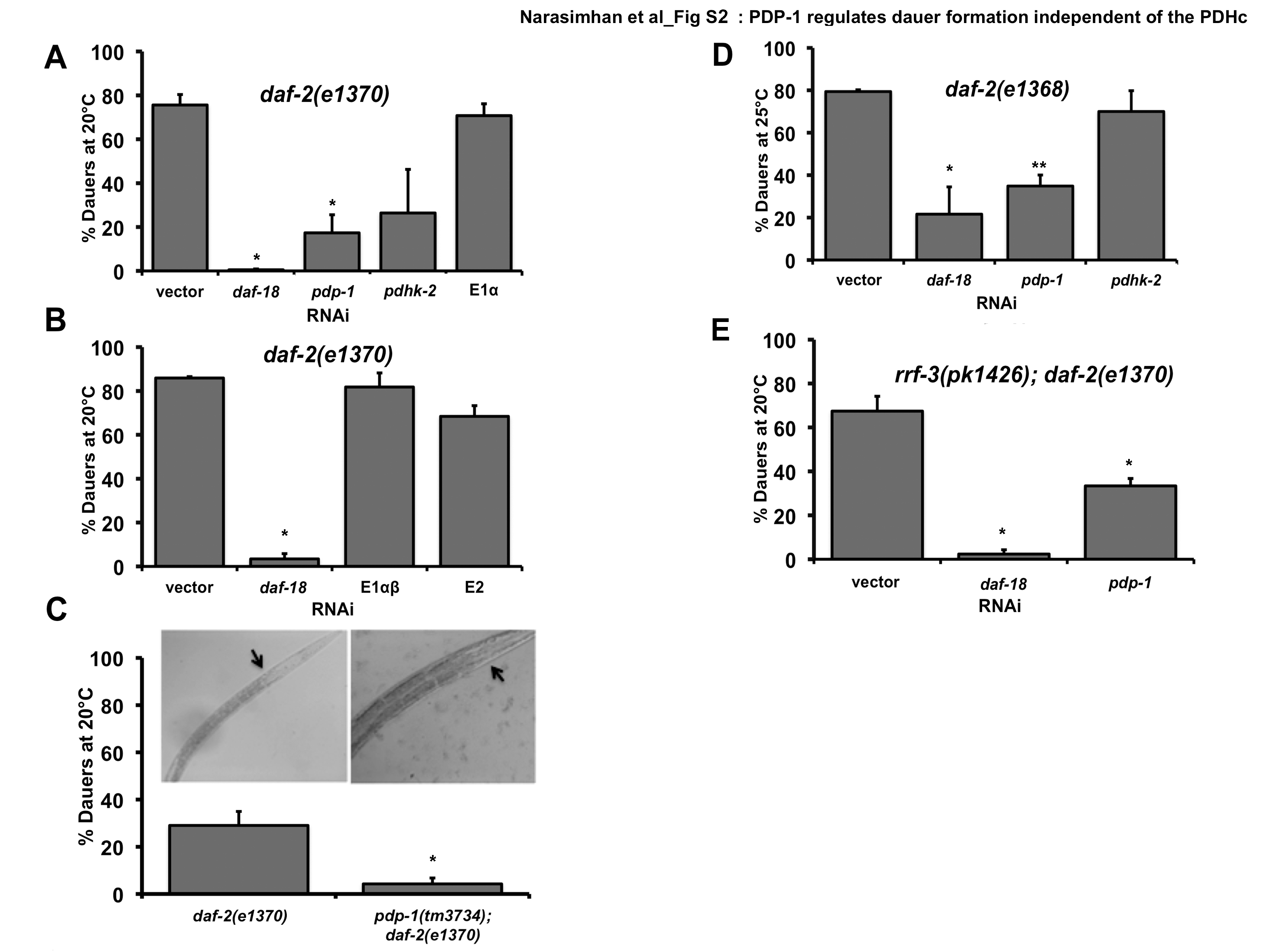

Supplement: Figure S2 — PDP-1 regulates dauer formation independent of the PDHc. Data shown are from one representative experiment. For the dauer assays, Error bars indicate the standard deviation among the different plates within one experiment. A) pdp-1 RNAi significantly suppresses daf-2(e1370) dauer formation (p<0.01), similar to daf-18 RNAi (p<0.01) while E1α RNAi has no effect. pdhk-2 RNAi results in a slight decrease in daf-2(e1370) dauer formation. B) Knockdown of components of the PDHc do not affect daf-2(e1370) dauer formation. RNAi of both, the E1α and E1β or the E2 subunit does not suppress dauer formation like daf-18 RNAi (p<0.01). C) A mutation in pdp-1 suppresses daf-2(e1370) dauer formation, similar to the effect of pdp-1 RNAi. (p<0.03). D) pdp-1 RNAi significantly suppresses daf-2(e1368) dauer formation (p<0.002) similar to daf-18 RNAi (p<0.007). pdhk-2 RNAi has no effect on daf-2(e1368) dauer formation. E) pdp-1 RNAi suppresses dauer formation in daf-2(e1370) mutants (p<0.02) in a RNAi-sensitized background, similar to daf-18 RNAi (p<0.02). (0.96 MB TIF) [file pgen.1001377.s002.tif]

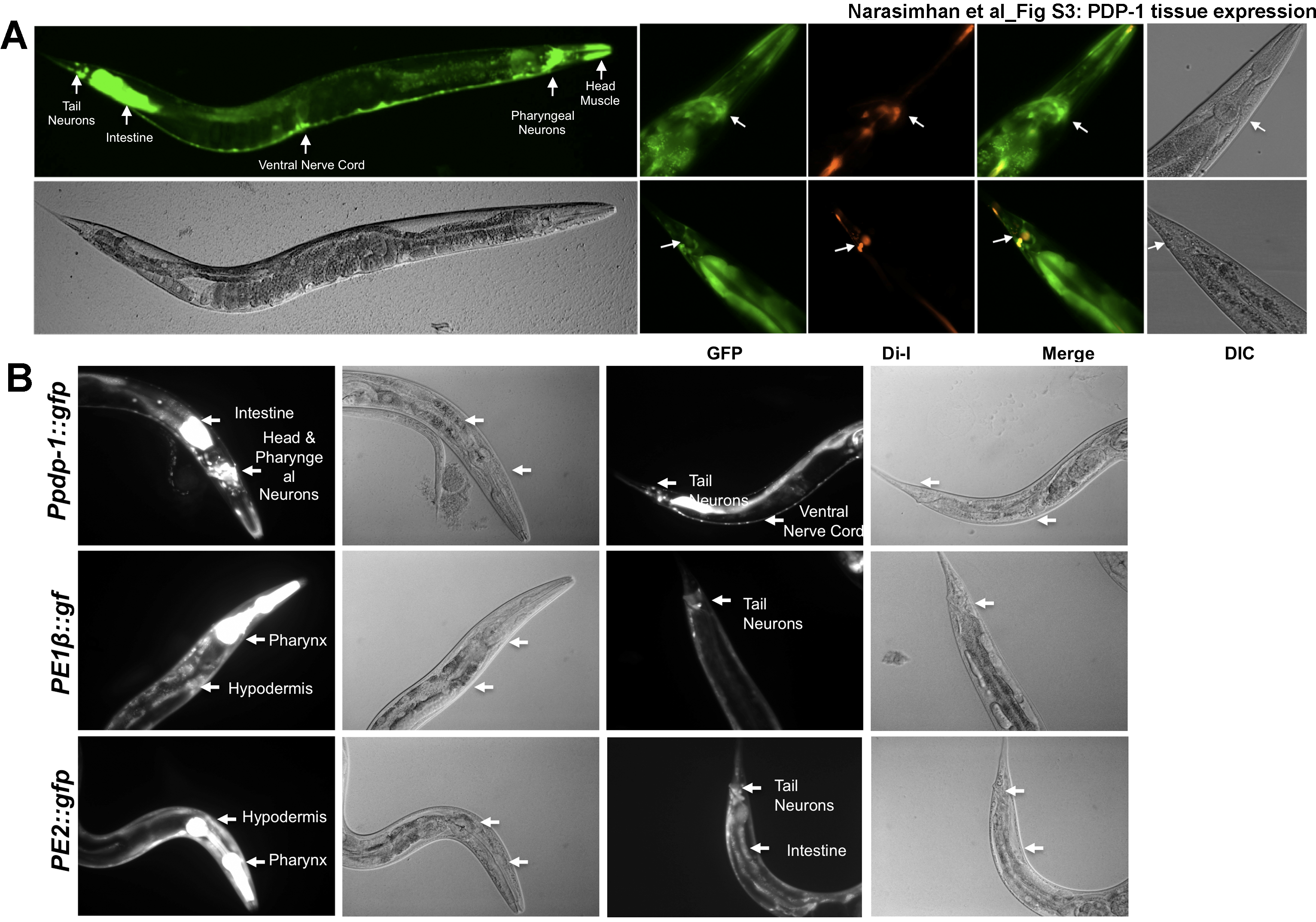

Supplement: Figure S3 — Tissue Expression patterns of PDP-1, A) Expression pattern of pdp-1 as visualized using a Ppdp-1::gfp transcriptional fusion strain. Di-I staining shows co-localization in amphid neurons. B) The Ppdp-1::gfp strain does not show complete overlap with the expression patterns of transcriptional fusion strains of the PDHc, PE1β::gfp and PE2::gfp. (4.41 MB TIF) [file pgen.1001377.s003.tif]

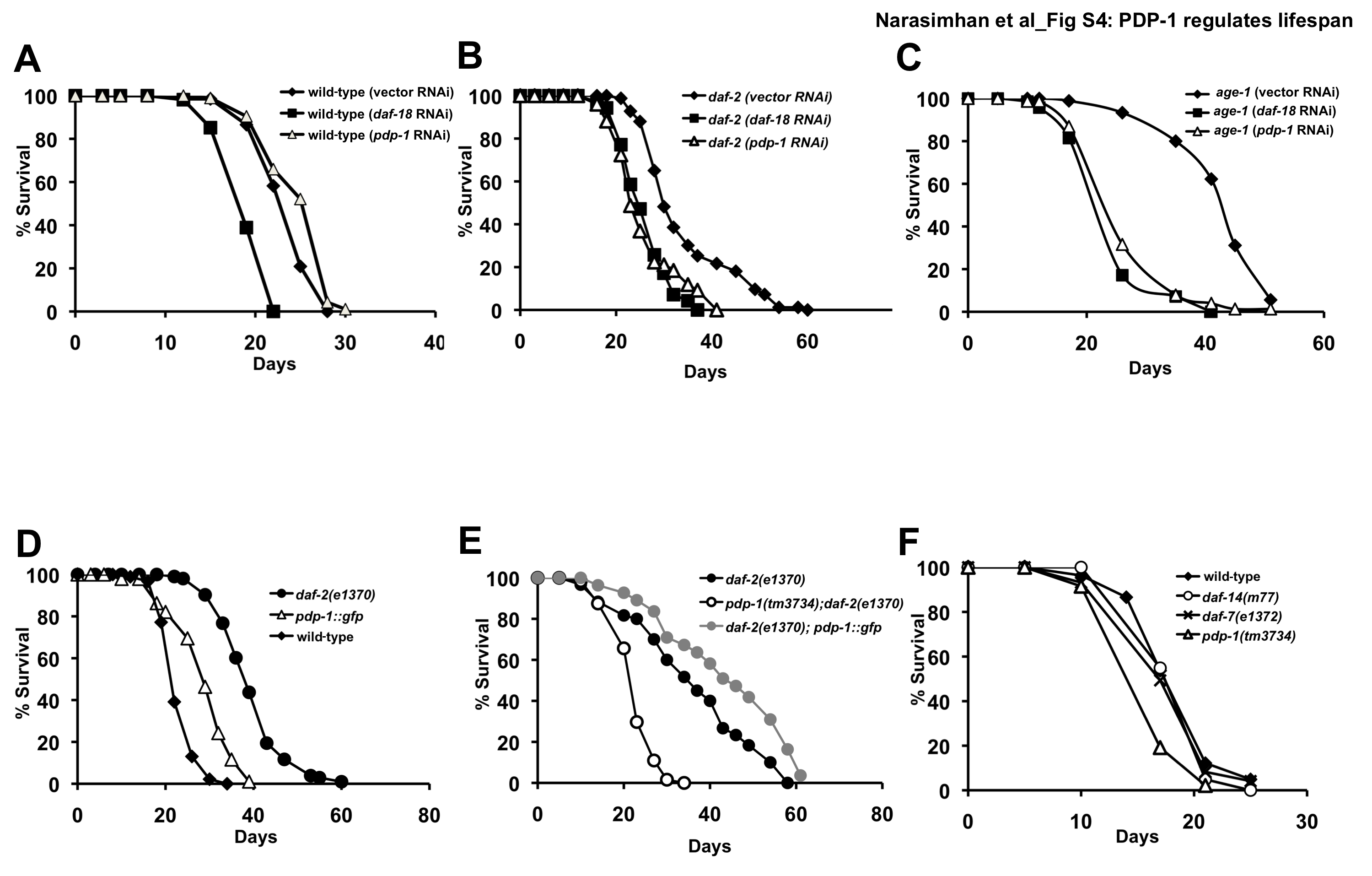

Supplement: Figure S4 — PDP-1 regulates lifespan. Data shown are from one representative experiment. A) pdp-1 RNAi does not significantly reduce the lifespan of wild-type worms (p<0.07). B) pdp-1 RNAi significantly reduces daf-2(e1370) lifespan (p<0.0001) similar to daf-18 RNAi (p<0.0001). C) pdp-1 RNAi significantly reduces age-1(hx546) lifespan (p<0.0001) similar to daf-18 RNAi (p<0.0001). D) Overexpression of pdp-1 increases lifespan (p<0.0001). E) Dosage modulation of pdp-1 can regulate daf-2 lifespan. pdp-1(tm3734); daf-2(e1370) worms live significantly shorter than daf-2(e1370) worms (p<0.0001) while daf-2(e1370); pdp-1::gfp worms live longer (p<0.0001). F) Mutations in daf-14 and daf-7 do not significantly increase lifespan. pdp-1(tm3734) mutants live shorter than wild-type worms (p<0.005). (0.62 MB TIF) [file pgen.1001377.s004.tif]

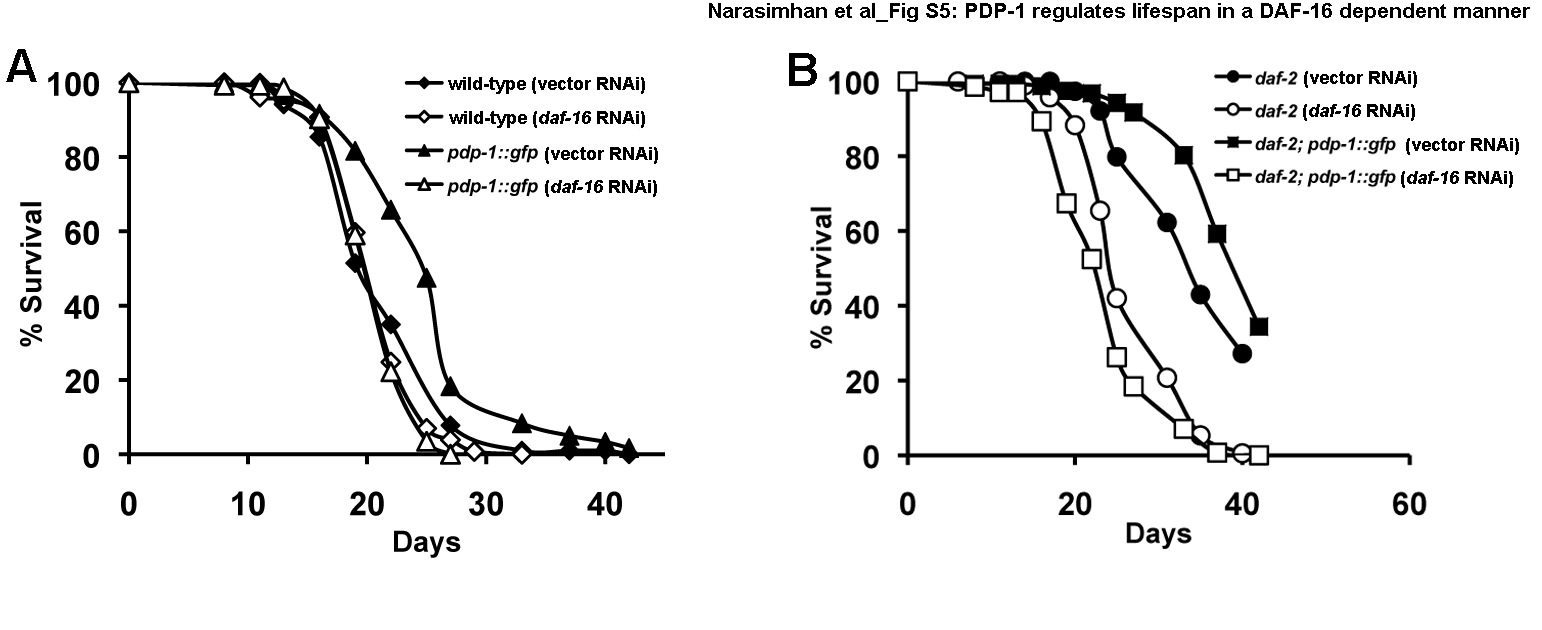

Supplement: Figure S5 — PDP-1 regulates lifespan in a DAF-16-dependent manner. A) Increased dosage of pdp-1 extends the lifespan of wild-type worms (p<0.005) and this extension is suppressed by daf-16 RNAi (p<0.0001). B) Increased dosage of pdp-1 further extends daf-2(e1370) lifespan (p<0.0001), and this extension is completely suppressed by daf-16 RNAi (p<0.0001). (0.13 MB TIF) [file pgen.1001377.s005.tif]

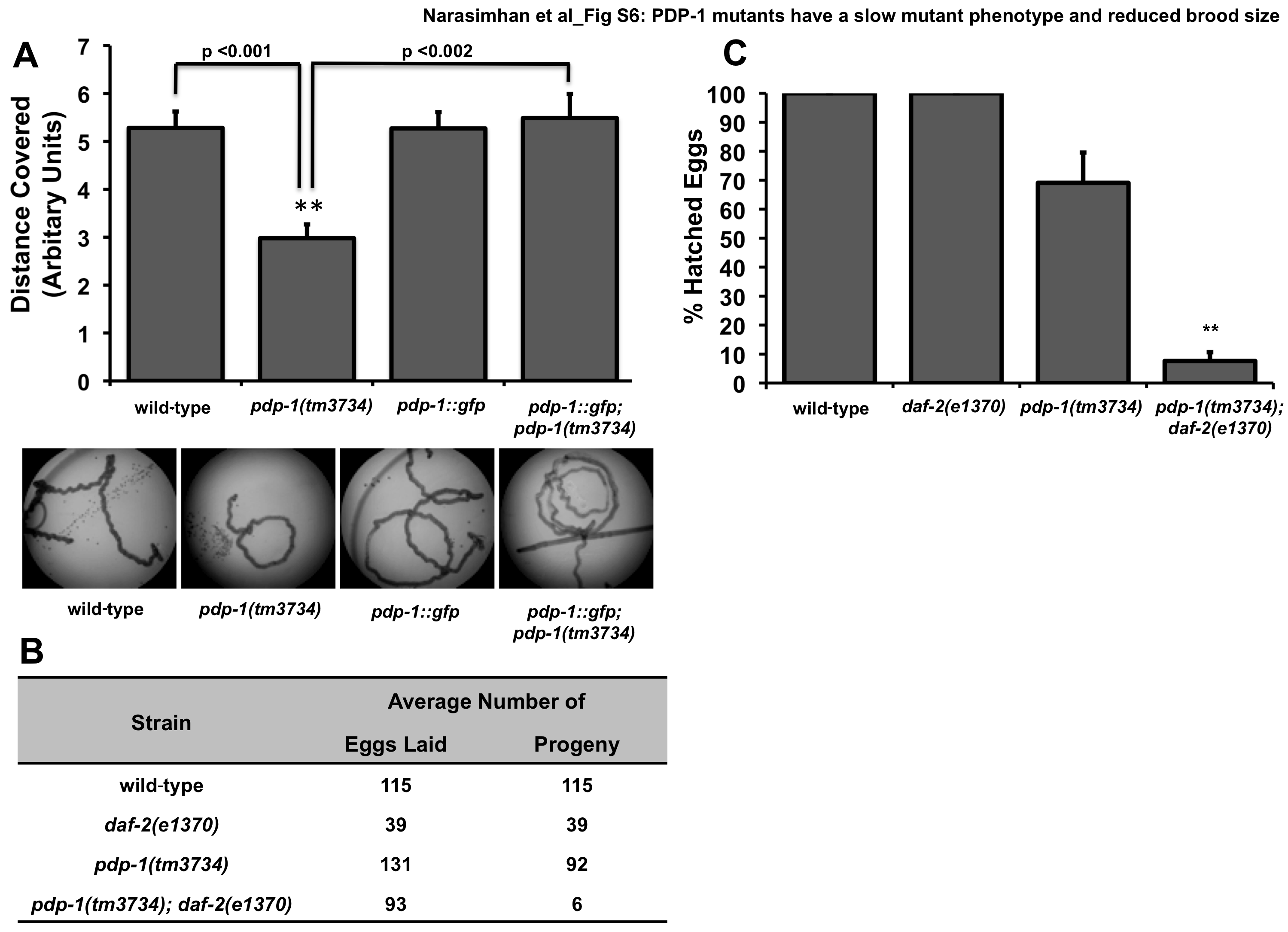

Supplement: Figure S6 — PDP-1 mutants have a slow movement phenotype and reduced brood size. Data shown are from one representative experiment. Error bars indicate the standard deviation among the different plates within one experiment. A) pdp-1(tm3734) mutants have a slow movement phenotype when compared to wild-type worms (p<0.001). This slow movement in the pdp-1(tm3734) mutant can be rescued by expression of a pdp-1::gfp transgene (p<0.002). Lower panel: Traces of wild-type, pdp-1(tm3734), pdp-1::gfp and pdp-1::gfp; pdp-1(tm3734) worms moving on a lawn of OP50. B) Brood size of wild-type, daf-2(e1370), pdp-1(tm3734) and pdp-1(tm3734); daf-2(e1370) animals as scored after 22.5 hours (total number of eggs laid) and 38 hours (total number of progeny). C) The % hatched eggs calculated from the number of progeny and number of eggs laid. pdp-1(tm3734) worms have fewer progeny (p<0.04) when compared to wild-type worms, however, this phenotype is far more severe in pdp-1(tm3734); daf-2(e1370) worms (p<0.005). (0.82 MB TIF) [file pgen.1001377.s006.tif]

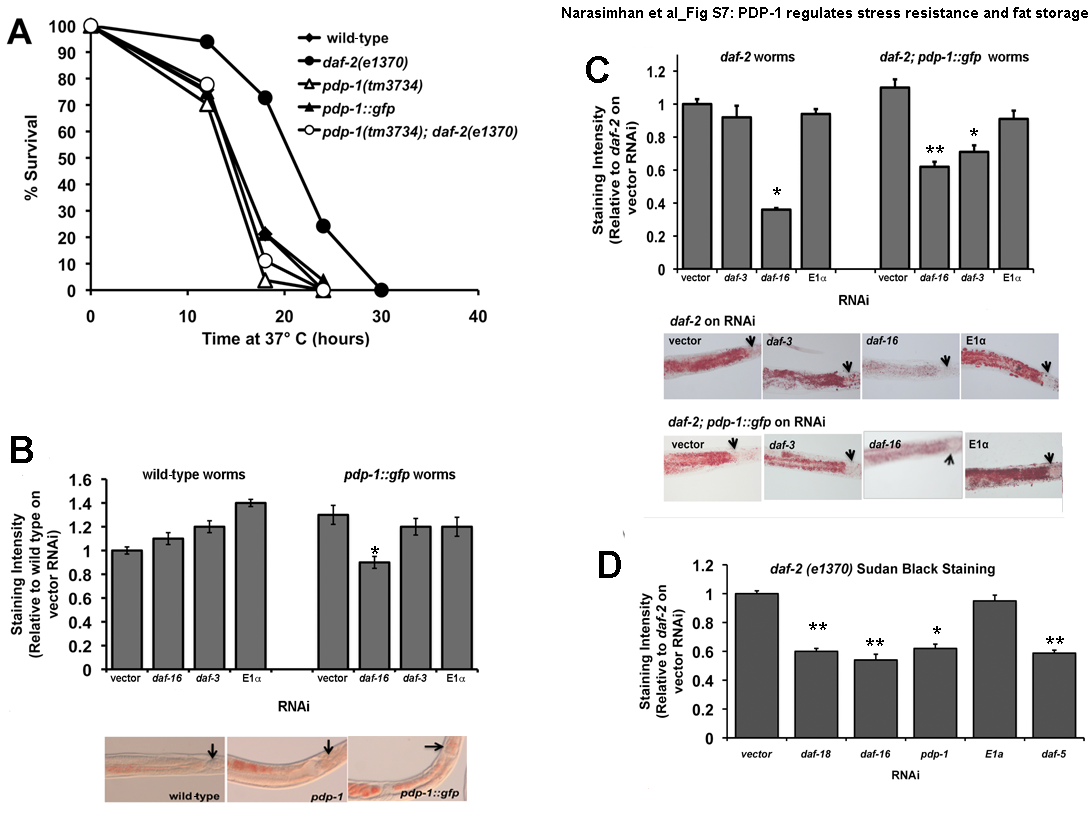

Supplement: Figure S7 — PDP-1 regulates stress resistance and fat storage. Data shown are from one representative experiment. Arrows indicate the lower bulb of the pharynx. A) PDP-1 regulates thermotolerance. A mutation in pdp-1 slightly reduces thermotolerance (p<0.06) of wild-type worms but significantly reduces daf-2(e1370) thermotolerance (p<0.05). B) Oil Red O Staining of adult worms. Top panel: Quantification of Oil Red O staining in wild-type and pdp-1::gfp worms. Overexpression of pdp-1 slightly enhances fat storage (p<0.01), and this enhancement is dependent on daf-16 RNAi (p<0.01) but not daf-3 or E1α RNAi. Lower panel: Oil Red O Staining of young adult worms showing comparable levels of fat between wild-type and pdp-1(tm3734) worms, while pdp-1::gfp young adults show slightly enhanced fat storage. C) Oil Red O Staining of daf-2(e1370) and daf-2(e1370); pdp-1::gfp worms. Top panel: Quantification of Oil Red O staining in daf-2(e1370) and daf-2; pdp-1::gfp worms. Similar to daf-2(e1370) worms, the fat storage of daf-2(e1370); pdp-1::gfp worms is suppressed by daf-16 RNAi (p<0.005) but not E1αRNAi. daf-3 RNAi slightly reduces the fat of daf-2; pdp-1::gfp but not daf-2(e1370) worms (p<0.01). Lower panel: Representative images of Oil Red O Staining in daf-2(e1370) and daf-2(e1370); pdp-1::gfp worms on daf-16, daf-3 and E1α RNAi. D) Quantification of Sudan Black Staining of daf-2(e1370) on different RNAi clones. The increased fat storage of daf-2(e1370) worms is suppressed on daf-18 (p<0.005), daf-16 (p<0.005), pdp-1 (p<0.007) and daf-5 RNAi (p<0.005). (0.36 MB TIF) [file pgen.1001377.s007.tif]

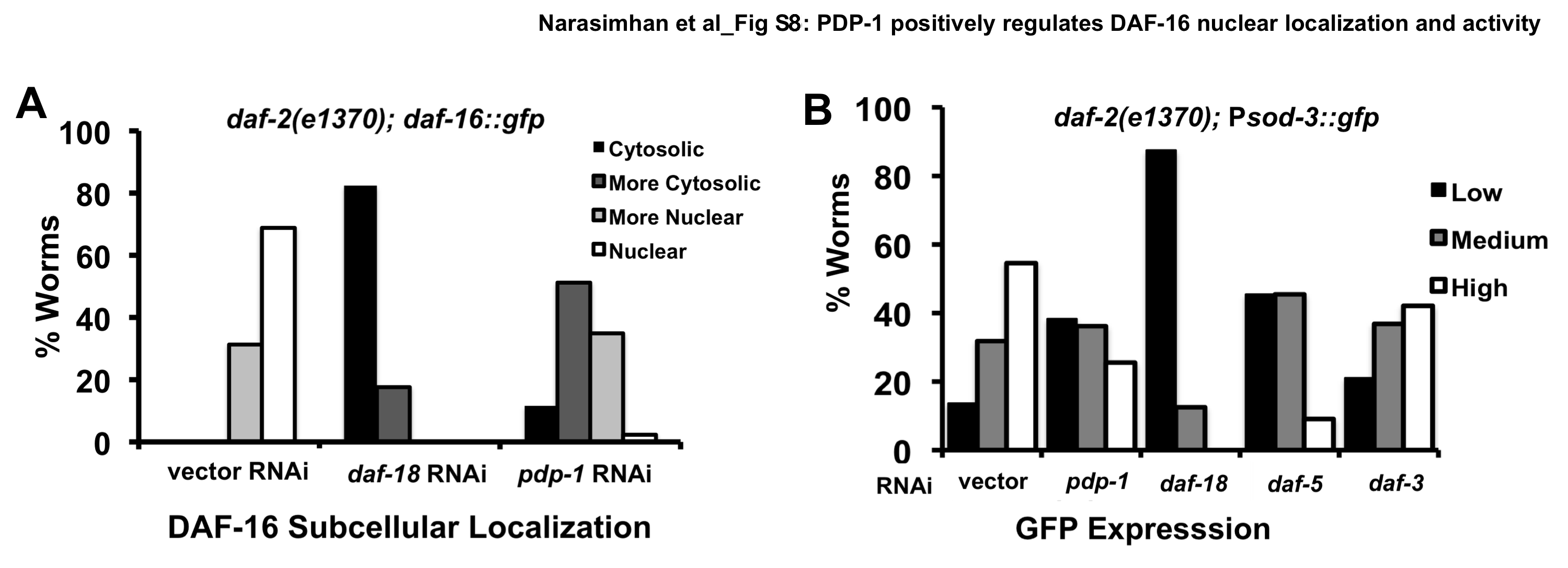

Supplement: Figure S8 — PDP-1 positively regulates DAF-16 nuclear localization and activity. A) Quantification of DAF-16 subcellular localization as observed in daf-2(e1370); daf-16::gfp worms on vector, daf-18 and pdp-1 RNAi. B) Quantification of GFP expression in a daf-2(e1370); Psod-3::gfp reporter strain grown on vector, daf-18, pdp-1, daf-5 and daf-3 RNAi. (0.47 MB TIF) [file pgen.1001377.s008.tif]

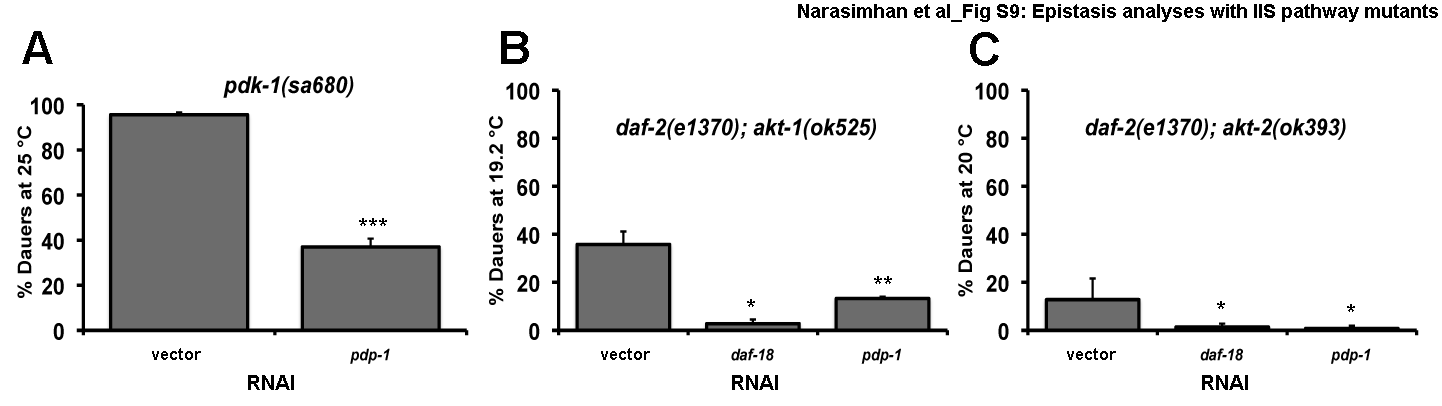

Supplement: Figure S9 — Epistasis analyses using mutants of the IIS pathway. A) pdp-1 RNAi significantly suppresses dauer formation of pdk-1(sa680) mutants (p<0.01). B) pdp-1 RNAi suppresses dauer formation of daf-2(e1370); akt-1(ok525) double mutants (p<0.03). C) pdp-1 RNAi suppresses dauer formation of daf-2(e1370); akt-2(ok393) double mutants (p<0.05). (0.07 MB TIF) [file pgen.1001377.s009.tif]

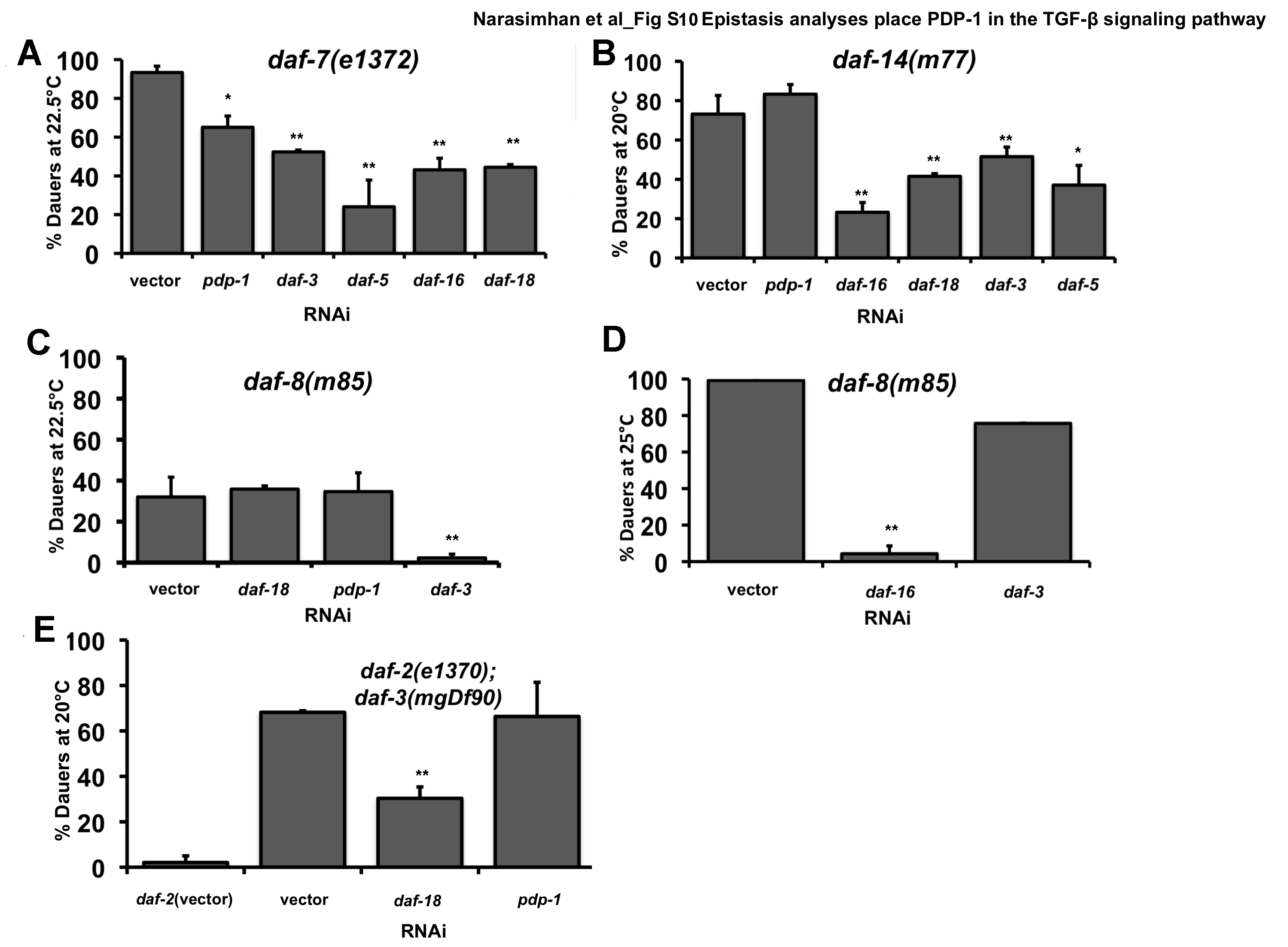

Supplement: Figure S10 — Epistasis analyses using mutants of the TGF-β pathway. A) daf-7(e1372) dauer formation is suppressed by pdp-1 RNAi (p<0.02), daf-18 RNAi (p<0.005), daf-16 RNAi (p<0.008) as well as the controls daf-3 RNAi (p<0.02) and daf-5 RNAi (p<0.05). B) pdp-1 RNAi has no effect on dauer formation of daf-14(m77) worms (p<0.1). However, daf-18 RNAi (p<0.05) and daf-16 RNAi (p<0.05) result in dauer suppression. C) pdp-1 RNAi has no effect on dauer formation of daf-8(m85) worms (p<0.3). Similarly daf-18 RNAi also has no effect on dauer formation (p<0.1). D) At 25°C daf-16 RNAi can robustly suppress dauer formation of daf-8(m85) worms (p<0.009), while daf-3 RNAi only has a partial effect (p<0.05). E) Dauer formation of daf-2(e1370); daf-3(mgDf90) is suppressed by daf-18 RNAi (p<0.04) but not pdp-1 RNAi (p<0.2). (0.70 MB TIF) [file pgen.1001377.s010.tif]

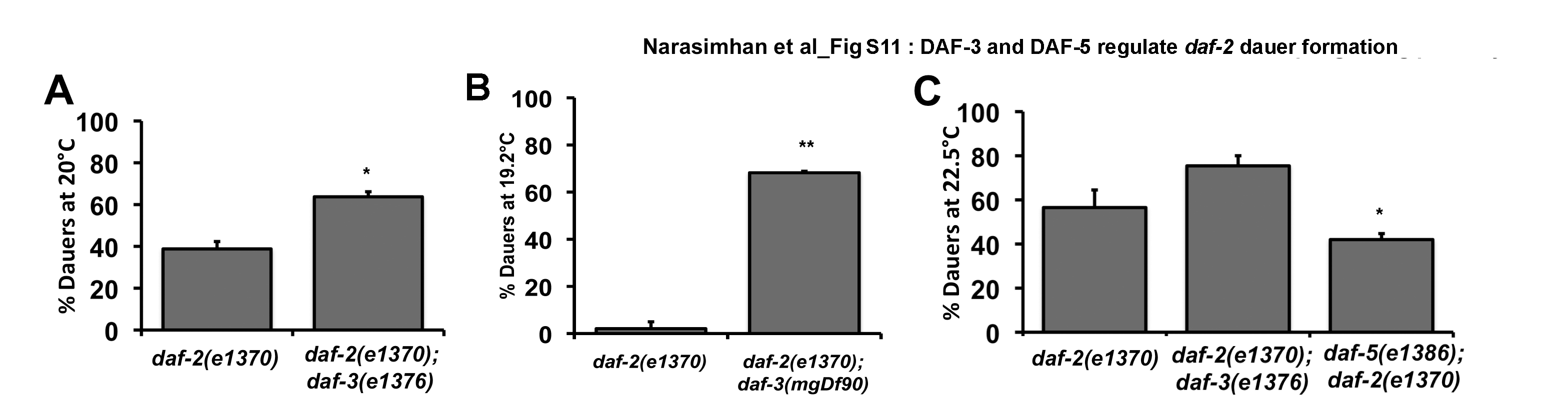

Supplement: Figure S11 — DAF-3 and DAF-5 regulate daf-2(e1370) dauer formation. Data shown are from one representative experiment. Error bars indicate the standard deviation among the different plates within one experiment. A) Dauer formation of daf-2(e1370); daf-3(e1376) double mutants is significantly enhanced over daf-2(e1370) worms (p<0.004). B) Dauer formation of daf-2(e1370); daf-3(mgDf90) double mutants is significantly enhanced over daf-2(e1370) worms (p<0.001). C) daf-3 (p<0.03) and daf-5 (p<0.06) mutations enhance and reduce daf-2 dauer formation. (0.32 MB TIF) [file pgen.1001377.s011.tif]

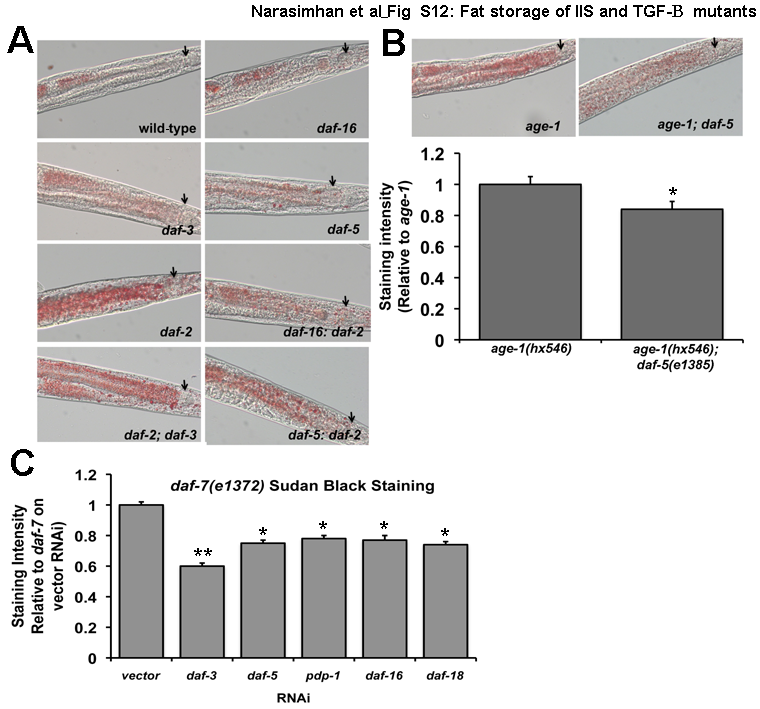

Supplement: Figure S12 — Crosstalk between the IIS and TGF-β signaling pathways in modulation of fat storage. Data shown are from one representative experiment. Error bars indicate the standard error among the different plates within one experiment. A) Oil Red O staining of single and double mutant adult worms of the IIS and TGF-β pathways. Arrows indicate the lower bulb of the pharynx. B) The increased fat storage of age-1(hx546) worms is suppressed by a mutation in daf-5. Top panel: Oil Red O staining of age-1(hx546) and age-1(hx546); daf-5(e1385) young adult worms. Arrows indicate the lower bulb of the pharynx. Lower panel: Quantification of Oil Red O staining shows significantly reduced fat in age-1(hx546); daf-5(e1385) worms as compared to the age-1(hx546) parental strain (p<0.009). C) Quantification of Sudan Black staining of daf-7(e1372) L3 worms on different RNAi bacteria. Fat storage of daf-7(e1372) animals is decreased by daf-3 (p<0.0001), daf-16 (p<0.001), pdp-1 (p<0.0001), daf-18 (p<0.0001) and daf-5 (p<0.0001) RNAi. (0.43 MB TIF) [file pgen.1001377.s012.tif]

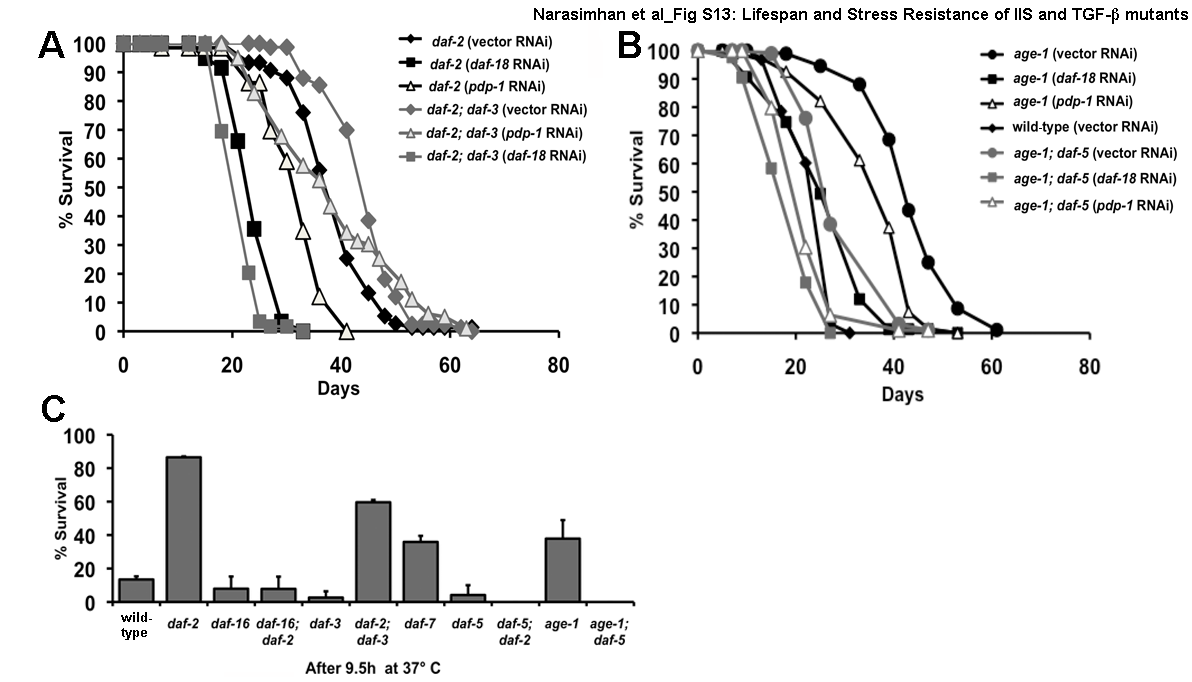

Supplement: Figure S13 — Crosstalk between the IIS and TGF-β signaling pathways in modulation of lifespan and stress resistance. Data shown for the lifespan assays are from one representative experiment. A) Lifespan of daf-2(e1370); daf-3(mgDf90) worms is enhanced over daf-2(e1370) mutants (p<0.001). pdp-1 RNAi can significantly suppress the lifespan of daf-2(e1370) worms (p<0.0001) but only has a partial effect on the lifespan of daf-2(e1370); daf-3(mgDf90) worms (p<0.01). daf-18 RNAi significantly reduces lifespan in both strains (p<0.0001). B) age-1(hx546); daf-5(e1385) double mutants live significantly shorter than age-1(hx546) worms (p<0.0001). Both pdp-1 and daf-18 RNAi significantly reduce the lifespan of both strains (p<0.0001). C) Survival of adult worms of the IIS and TGF-β pathways after 9.5 hours at 37°C. Data shown is an average of two independent repeats, with error bars indicating the variation between two repeats. (0.20 MB TIF) [file pgen.1001377.s013.tif]

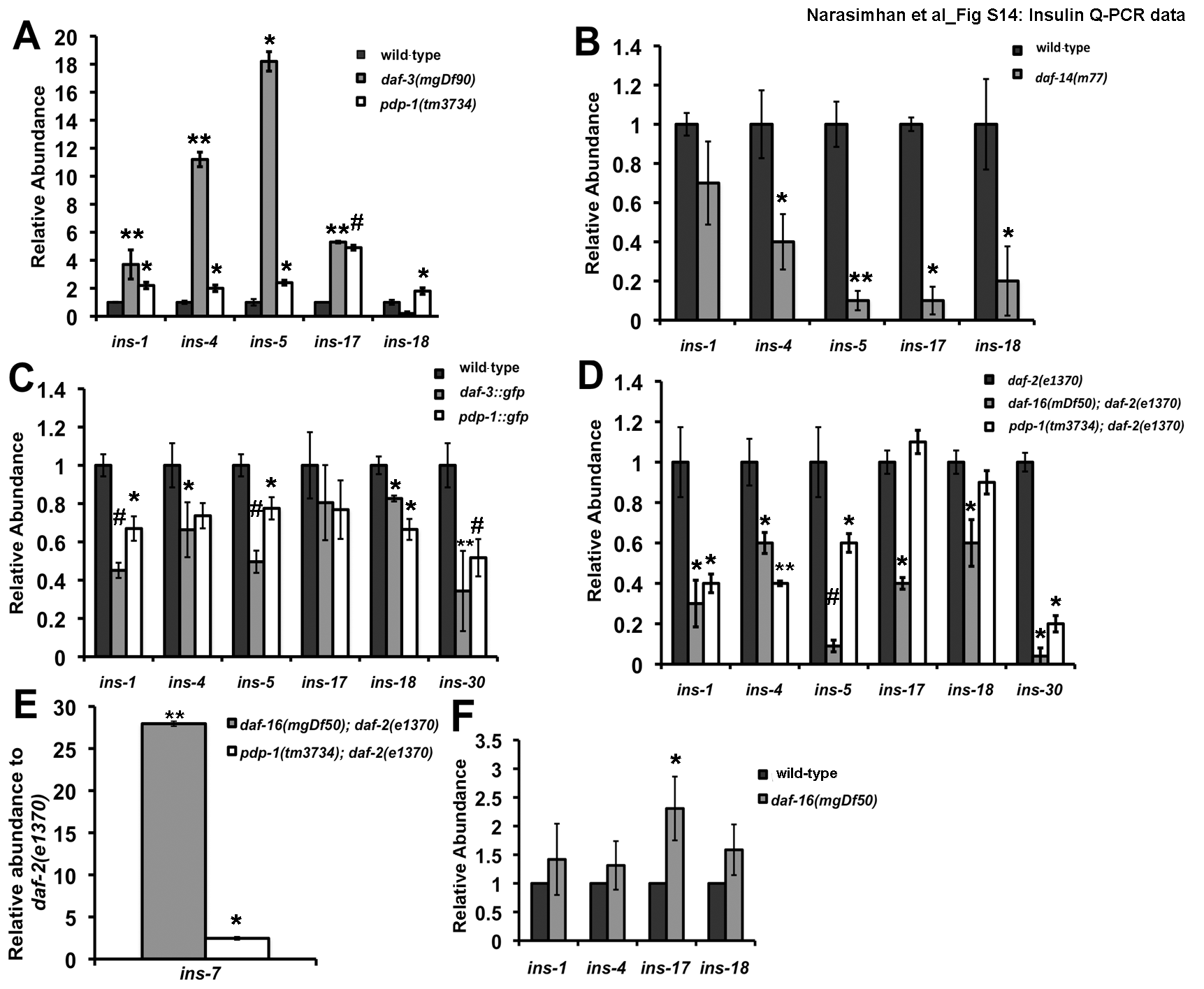

Supplement: Figure S14 — Q-PCR experiments. Data shown are from one representative experiment. Error bars represent standard error of the mean within triplicates. A) The levels of several insulin genes are elevated in daf-3(mgDf90) and pdp-1(tm3734) worms. * p<0.05, **p<0.02, #p<0.0009. B) The levels of the same set of insulins are markedly decreased in daf-14(m77) worms. *p<0.05, **p<0.0007. C) Insulins levels are significantly decreased in daf-3::gfp and pdp-1::gfp strains. *p<0.05, **p<0.01, #p<0.001. D) Insulin gene regulation is under the control of the IIS pathway. Compared to daf-2(e1370) worms, the levels of several insulins change in pdp-1(tm3734); daf-2(e1370) worms and daf-16(mgDf50); daf-2(e1370) worms. *p<0.05, **p<0.0007, #p<0.0005. E) ins-7 levels are significantly increased in daf-16(mgDf50); daf-2(e1370) and pdp-1(tm3734); daf-2(e1370) double mutants, compared to daf-2(e1370). *p<0.04, **p<0.001. F) The levels of several insulin genes are unchanged in daf-16(mgDf50) single mutants. *p<0.05. (0.58 MB TIF) [file pgen.1001377.s014.tif]

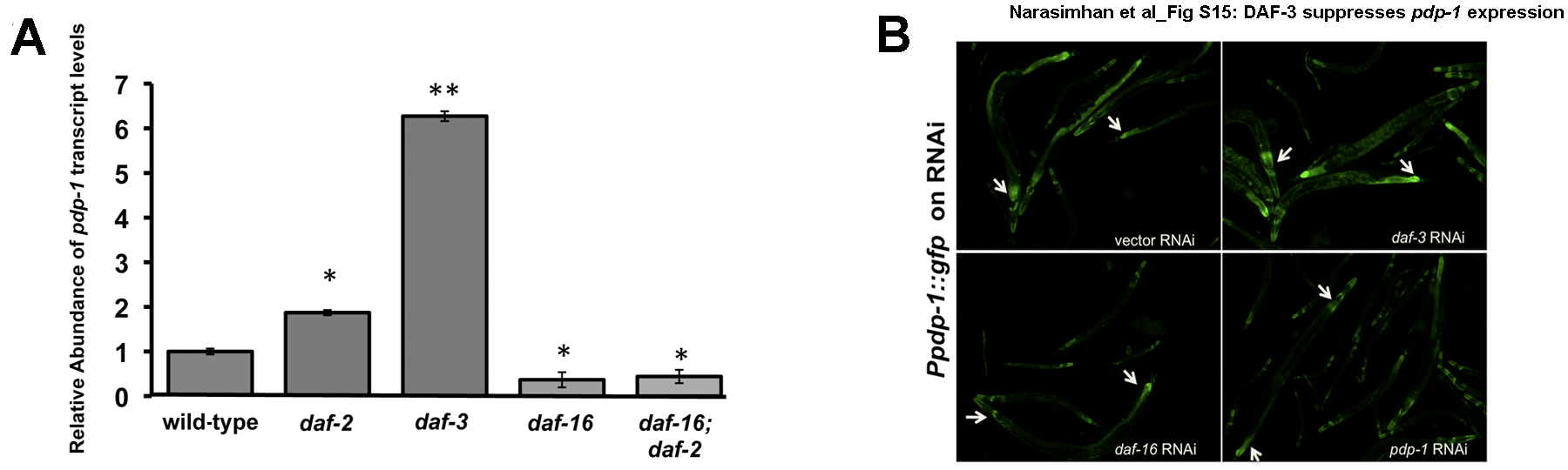

Supplement: Figure S15 — DAF-3 regulates pdp-1 expression. A) Q-PCR results showing elevated levels of pdp-1 in daf-3(mgDf90) mutants. Also, expression is slightly increased over wild-type worms in daf-2(e1370) mutants but decreased in daf-16(mgDf50) worms as well as daf-16(mgDf50); daf-2(e1370) worms. Data shown are from one representative experiment. Error bars represent standard error of the mean within triplicates. B) Compared to vector RNAi, GFP expression of the Ppdp-1::gfp transcriptional fusion strain is higher on daf-3 RNAi, and slightly reduced on daf-16 RNAi (100× magnification). (0.30 MB TIF) [file pgen.1001377.s015.tif]
