# Supplementary material for: PDP-1 Links the TGF-β and IIS Pathways to Regulate Longevity, Development, and Metabolism
Source: PLoS Genet. 2011 Apr 21;7(4):e1001377. doi: 10.1371/journal.pgen.1001377 (PMC3080858; doi:10.1371/journal.pgen.1001377)
Supplement: Table S1 — Lifespans of IIS and TGF-β pathway mutants. (0.04 MB DOC) [file pgen.1001377.s016.doc]

| **Strain** | **Mean Lifespan at 15°C  ± Std. Dev Days** | **n** |
| --- | --- | --- |
| **wild-type** | **22.1 ± 3.6** | **72** |
| ***daf-2(e1370)*** | **31.7 ± 5.4** | **73** |
| ***daf-16(mgDf50)*** | **17.8 ± 3.0** | **73** |
| ***daf-16(mgDf50); daf-2(e1370)*** | **19.3 ± 2.3** | **76** |
| ***daf-7(e1372)*** | **23.0 ± 3.9** | **49** |
| ***daf-3(mgDf90)*** | **18.5 ± 1.9** | **74** |
| ***daf-2(e1370); daf-3(mgDf90)*** | **31.1 ± 11.4** | **55** |
| **Strain** | **Mean Lifespan at 20°C  ± Std. Dev Days** | **n** |
| **wild-type** | **21.1 ± 1.8** | **72** |
| ***daf-2(e1370)*** | **36.7 ± 6.5** | **59** |
| ***daf-16(mgDf50)*** | **15.5 ± 1.3** | **58** |
| ***daf-16(mgDf50); daf-2(e1370)*** | **16.1 ± 1.3** | **54** |
| ***daf-3(mgDf90)*** | **14.6 ± 2.7** | **55** |
| ***daf-2(e1370); daf-3(mgDf90)*** | **47.6 ± 8.2** | **55** |
| **Strain** | **Mean Lifespan at 25°C**  **± Std. Dev Days** | **n** |
| **wild-type** | **11.3 ± 1.8** | **62** |
| ***daf-7(e1372)*** | **12.6 ± 2.9** | **35** |
| ***daf-2(e1370)*** | **19.7 ± 4.9** | **61** |
| ***daf-2(e1370); daf-3(mgDf90)*** | **24.5 ± 2.3** | **60** |
| ***daf-5(e1386); daf-2(e1370)*** | **8.5 ± 1.0** | **51** |

**Supplementary Table 1: Lifespans of IIS and TGF-β pathway mutants**
