# Supplementary material for: PDP-1 Links the TGF-β and IIS Pathways to Regulate Longevity, Development, and Metabolism
Source: PLoS Genet. 2011 Apr 21;7(4):e1001377. doi: 10.1371/journal.pgen.1001377 (PMC3080858; doi:10.1371/journal.pgen.1001377)
Supplement: Table S2 — List of insulins tested in this manuscript. (0.04 MB DOC) [file pgen.1001377.s017.doc]

Supplementary Table 2: List of insulins tested in this manuscript

| **Insulin** | **Lifespan or Dauer Phenotype in** | |
| --- | --- | --- |
|  | **Mutants/RNAi** | **Overexpression** |
| ***ins-1*** | **No phenotype**^a^*** | ***ins-1*  overexpression enhances dauer arrest of *daf-2* and *daf-7* mutants and extends the lifespan of wild-type worms *^a^*** |
| ***ins-4*** | **No phenotype*** | ***ins-4* overexpression suppresses dauer formation of TGF-β pathway mutants *^b,c^*** |
| ***ins-5*** | **No phenotype*** | **nt** |
| ***ins-7*** | ***ins-7* RNAi extends the lifespan of wild-type worms and enhances *daf-2(e1370)* dauer arrest *^d,e^*** | ***ins-7* overexpression in the intestine reduces the lifespan of wild-type worms *^e^*** |
| ***ins-17*** | **Sick/Sterile*** | **nt** |
| ***ins-18*** | **1. *ins-18* RNAi extends the lifespan of wild-type worms*^f^* but slightly reduces the lifespan of other mutants *^d^***  **2.The *ins-18(tm339)* mutation reduces *daf-7* dauer arrest *^g^*** | ***ins-18* overexpression enhances dauer formation of wild-type worms and weak *daf-2* mutants *^a^*** |
| ***ins-30*** | ***ins-30* RNAi slightly extends the lifespan of *daf-2(e1370)* mutants *^h^*** | **nt** |
| ***ins-33*** | ***ins-33* mutation/RNAi results in embryonic lethality/slow growth**^i^*** | **nt** |
| ***ins-35*** | **No phenotype*** | **nt** |
| ***daf-28*** | **The *daf-28(sa191)* mutation induces dauer arrest and slightly extends lifespan *^b,j^*** | ***daf-28* overexpression suppresses dauer formation of TGF-β pathway mutants *^b,c^*** |
