# Supplementary material for: PDP-1 Links the TGF-β and IIS Pathways to Regulate Longevity, Development, and Metabolism
Source: PLoS Genet. 2011 Apr 21;7(4):e1001377. doi: 10.1371/journal.pgen.1001377 (PMC3080858; doi:10.1371/journal.pgen.1001377)
Supplement: Table S3 — Summary of trends observed in the Q-PCR Experiments. (0.05 MB DOC) [file pgen.1001377.s018.doc]

**Supplementary Table 3: Summary of trends observed in the Q-PCR Experiments**

| **Trend relative to wild-type worms** | | | | | |  | **Trend relative to *daf-2(e1370)* worms** | | |
| --- | --- | --- | --- | --- | --- | --- | --- | --- | --- |
| **Insulin** | ***daf-3***  ***(mgDf90)*** | ***pdp-1***  ***(tm3734)*** | ***daf-14***  ***(m77)*** | ***daf-3::gfp*** | ***pdp-1::gfp*** |  | **Insulin** | ***daf-16(mgDf50)***  ***; daf-2(e1370)*** | ***pdp-1(tm3734); daf-2(e1370)*** |
| ***ins-1*** | **increased** | **increased** | **decreased** | **decreased** | **no change** |  | ***ins-1*** | **decreased** | **decreased** |
| ***ins-4*** | **increased** | **increased** | **decreased** | **decreased** | **decreased** |  | ***ins-4*** | **decreased** | **decreased** |
| ***ins-5*** | **increased** | **increased** | **decreased** | **decreased** | **decreased** |  | ***ins-5*** | **decreased** | **decreased** |
| ***ins-17*** | **increased** | **increased** | **decreased** | **no change** | **no change** |  | ***ins-7*** | **increased** | **increased** |
| ***ins-18*** | **no change** | **no change** | **decreased** | **decreased** | **decreased** |  | ***ins-17*** | **decreased** | **no change** |
| ***ins-7^#^*** |  | | | | |  | ***ins-18*** | **no change** | **no change** |
| ***ins-30*** | **no change** | **no change** | **no change** | **decreased** | **decreased** |  | ***ins-30*** | **decreased** | **decreased** |
| ***ins-33*** | **nd** | **nd** | **nd** | **nd** | **nd** |  | ***ins-33*** | **nd** | **nd** |
| ***ins-35*** | **nd** | **nd** | **nd** | **nd** | **nd** |  | ***ins-35*** | **nd** | **nd** |
| ***daf-28^a^*** | **nd** | **variable** | **variable** | **nd** | **variable** |  | ***daf-28*** | **decreased** | **variable*^a^*** |
