# Supplementary material for: PDP-1 Links the TGF-β and IIS Pathways to Regulate Longevity, Development, and Metabolism
Source: PLoS Genet. 2011 Apr 21;7(4):e1001377. doi: 10.1371/journal.pgen.1001377 (PMC3080858; doi:10.1371/journal.pgen.1001377)
Supplement: Table S4 — List of strains used in this manuscript. (0.06 MB DOC) [file pgen.1001377.s019.doc]

|  | **Strain** | **Strain #** | **Additional information** |
| --- | --- | --- | --- |
| **1.** | **wild-type (N2)** |  |  |
| **2.** | ***daf-2(e1370)*** | **CB1370** |  |
| **3.** | ***daf-2(e1368)*** | **DR1572** |  |
| **4.** | ***pdp-1(tm3734)*** | **HT1666** | **Backcrossed to N2 4x** |
| **5.** | ***pdp-1(tm3734); daf-2(e1370)*** | **HT1819** |  |
| **6.** | ***Ppdp-1::gfp*** | **HT1856** | **Contains the *unc-119* rescue gene** |
| **7.** | ***age-1(hx546)*** | **TJ1052** |  |
| **8.** | ***pdp-1::gfp*** | **HT1857** | **Contains the *unc-119* rescue gene**  **Backcrossed to N2 4x** |
| **9.** | ***daf-2(e1370); pdp-1::gfp*** | **HT1581** |  |
| **10.** | ***daf-2(e1370); daf-16::gfp*** | **HT1531** | **Padmanabhan et al, 2009**  **Overexpression of the DAF-16a isoform** |
| **11.** | ***daf-2(1370); Psod-3::gfp*** | **HT1643** | **Padmanabhan et al, 2009** |
| **12.** | ***daf-16(mgDf50); daf-2(e1370)*** | **HT1858** | **Kwon, et al, 2010** |
| **13.** | ***pdk-1(sa680)*** | **JT9609** |  |
| **14.** | ***daf-2(e1370); akt-1(ok595)*** | **HT1547** | **Padmanabhan et al, 2009** |
| **15.** | ***daf-2(e1370); akt-2(ok393)*** | **HT1548** | **Padmanabhan et al, 2009** |
| **16.** | ***dpy-5(e907); sIs11033*** | **BC12915** | **Transcriptional fusion of the gene F23B12.5 (E2 subunit of PDH)**  **McKay et. al, 2004** |
| **17.** | ***dpy-5(e907); sIs13981*** | **BC14524** | **Transcriptional fusion of the gene C04C3.3 (E1β subunit of PDH)**  **McKay et. al, 2004** |
| **18.** | ***daf-7(e1372)*** | **CB1372** |  |
| **19.** | ***daf-14(m77)*** | **DR77** | **Inoue et al., 2000** |
| **20.** | ***daf-8(m85)*** | **DR1631** |  |
| **21.** | ***daf-2(e1370); daf-3(mgDf90)*** | **HT1607** | **Padmanabhan et al, 2009** |
| **22.** | ***daf-2(e1370); daf-3(e1376)*** | **HT1608** |  |
| **23.** | ***daf-3(e1376)*** | **CB1376** |  |
| **24.** | ***daf-3(mgDf90)*** | **GR1311** | **Patterson et al, 1997** |

**Supplementary Table 4: List of strains used in this manuscript**
