# Supplementary material for: PDP-1 Links the TGF-β and IIS Pathways to Regulate Longevity, Development, and Metabolism
Source: PLoS Genet. 2011 Apr 21;7(4):e1001377. doi: 10.1371/journal.pgen.1001377 (PMC3080858; doi:10.1371/journal.pgen.1001377)
Supplement: Table S5 — List of primers used in this manuscript. (0.13 MB DOC) [file pgen.1001377.s020.doc]

**Supplementary Table 5: List of primers used in this manuscript**

| **Primer name** | **Gene** | **Sequence (5’-3’)** | **Used For** |
| --- | --- | --- | --- |
| **L6ORF FW2** | ***pdp-1*** | **CGTACCTTTGTGCTTCAGTG** | **Detection of the 505 bp deletion in *pdp-1(tm3734)* mutants**  **wild-type: 1110 bp, *pdp-1(tm3734)*: 605 bo** |
| **L6ORF RV2** | ***pdp-1*** | **GTTCACCTCTTGCTCGATCT** |
| **L6POFW1** | ***pdp-1*** | **TGCAATTTTTCGGGGTTTTA** | **Verifying the presence of the *pdp-1* promoter and coding region in translational fusion constructs** |
| **L6PORV1** | ***pdp-1*** | **GCTGAAGAGCCATTCGAGTC** |
| **OG FW1** | ***pdp-1* and *gfp*** | **GGGAGAACTTTTTCCGCTTC** | **Checking the *pdp-1* coding region fused to the *gfp* gene in translational fusion constructs** |
| **OG RV1** | ***pdp-1* an*d gfp*** | **TCGAGAAGCATTGAACACCA** |
| **ins-1 Fw** | ***ins-1*** | **GACGCATCGA TTCGACTATG** | **Q-PCR** |
| **ins-1 Rv** | ***ins-1*** | **GGTCGGCGGAACGTTTGAAA** |
| **ins-4 Fw** | ***ins-4*** | **CCGAGCACGA CGAGTCCCAG** | **Q-PCR** |
| **ins-4 Rv** | ***ins-4*** | **CCATGTCCTCTTGTGGCGTG** |
| **ins-5 Fw** | ***ins-5*** | **GCGATCTCAA GAGCCGACCG** | **Q-PCR** |
| **ins-5 Rv** | ***ins-5*** | **CGATTCCGTTTTGTGGTTGG** |
| **ins-7 Fw** | ***ins-7*** | **CATG CGAATCGAATACTGAA** | **Q-PCR** |
| **ins-7 Rv** | ***ins-7*** | **CACTGTTTTCGAATGAAGTC** |
| **ins-17 Fw** | ***ins-17*** | **CT GTAGCCGCAT TCGGGCTG** | **Q-PCR** |
| **ins-17 Rv** | ***ins-17*** | **ATGAGGCACCACCTGGTGGG** |
| **ins-18 Fw** | ***ins-18*** | **ATGTGCCCA CCAGGTGGTT C** | **Q-PCR** |
| **ins-18 Rv** | ***ins-18*** | **CAGAGCACGTTTTTCGAAATAT** |
| **ins-30 Fw** | ***ins-30*** | **GCTCAGGGAG CCAAGAAGAC** | **Q-PCR** |
| **ins-30 Rv** | ***ins-30*** | **TTTCGTGGAGGTCATCAGCG** |
| **ins-33 Fw** | ***ins-33*** | **GTCATCGCCA TCATGGCCAG** | **Q-PCR** |
| **ins-33 Rv** | ***ins-33*** | **TGAGGAGAGTATCATCAGAA** |
| **ins-35 Fw** | ***ins-35*** | **GGAATCAAT AATCGTCACT G** | **Q-PCR** |
| **ins-35 Rv** | ***ins-35*** | **CCATGAGAATCTTTTCATAG** |
| **daf-28 Fw** | ***daf-28*** | **GTTCCAGGTG TGGCCGTGAG** | **Q-PCR** |
| **daf-28 Rv** | ***daf-28*** | **CAATTCCTTCTTGTGGTTCA** |
| **sod-3-52F** | ***sod-3*** | **GGAGTTCTCGCCGTCCG** | **Q-PCR** |
| **sod-3-102R** | ***sod-3*** | **GTCGAATGGGAGATCTGGGAG** |
| **hsp-12.6 fw** | ***hsp-12.6*** | **TGGAGTTGTCAATGTCCTCG** | **Q-PCR** |
| **hsp12.6 Rv** | ***hsp-12.6*** | **GACTTCAATCTCTTTTGGGAGG** |
| **Sod-5 Fw** | ***sod-5*** | **CCACAGGACGTTGTTTCCAA** | **Q-PCR** |
| **Sod-5 Rv** | ***sod5*** | **ACCTTCGGCTTTCTGGGTAA** |
| **scl-1 fw** | ***scl-1*** | **CAATCAAGCATTGTGGATGC** | **Q-PCR** |
| **scl-1 rv** | ***scl-1*** | **GGAATCCACGACCATTTTCC** |
| **Cpr-2 Fw** | ***cpr-2*** | **CTGCGTAAACCTTCAAACTC** | **Q-PCR** |
| **Cpr-2 Rv** | ***cpr-2*** | **ATGCGGAGTTACCATAGTTC** |
| **pdp-1utrFw1** | ***pdp-1*** | **aatgccatgcacacattttt** | **Q-PCR for verifying RNAi knockdown** |
| **pdp-1 utrRv1** | ***pdp-1*** | **ggggagactctttggcataa** |
| **daf-16rtfw** | ***daf-16*** | **aacttcaagccaatgccact** | **Q-PCR for verifying RNAi knockdown** |
| **daf-16rtrv** | ***daf-16*** | **tccaccattttgatagtttcca** |
| **pdhk-2Fw2** | ***pdhk-2*** | **cctttggctggttacggata** | **Q-PCR for verifying RNAi knockdown** |
| **pdhk-2Rv2** | ***pdhk-2*** | **cgagcgtatagtcgagatagtgg** |
| **daf-3Fw2** | ***daf-3*** | **cgtcaattcatgattgtccagt** | **Q-PCR for verifying RNAi knockdown** |
| **daf-3Rv2** | ***daf-3*** | **cgtaggcaatgttgattttca** |
| **daf-5rtFw** | ***daf-5*** | **tttgcttcgctacaaaaacg** | **Q-PCR for verifying RNAi knockdown** |
| **daf-5rtRv** | ***daf-5*** | **ctccatgactgctcggaact** |
| **daf-18rtFw** | ***daf-18*** | **gcagagaggctaaggaatgg** | **Q-PCR for verifying RNAi knockdown** |
| **daf-18rtRv** | ***daf-18*** | **cagctattacatgtttatcgtctgc** |
| **E1ArtFw** | **T05H10.6** | **ggccatgaaataccgtgaac** | **Q-PCR for verifying RNAi knockdown** |
| **E1ArtRv** | **T05H10.6** | **gcctcgaaaagttgtccttg** |
| **E1bFw2** | **C04C3.3** | **aagtgtggaaacctgttcgtg** | **Q-PCR for verifying RNAi knockdown** |
| **E1bRv2** | **C04C3.3** | **attgtggaagccgctctg** |
| **E2Fw2** | **F23B12.5** | **cggaggagcatctgataagc** | **Q-PCR for verifying RNAi knockdown** |
| **E2Rv2** | **F23B12.5** | **cttcttatatccttcagcctcgtc** |
| **E3Fw2** | **LLC1.3** | **gttgaaacaggagggagtcg** | **Q-PCR for verifying RNAi knockdown** |
| **E3Rv2** | **LLC1.3** | **agagttggcgacgaatgg** |
| **acts-5** | **actin** | **CTCTTGCCCCATCAACCATG** | **Q-PCR** |
| **acts-3** | **actin** | **CTTGCTTGGAGATCCACATC** |
